# Artemether–lumefantrine–amodiaquine or artesunate–amodiaquine combined with single low-dose primaquine to reduce *Plasmodium falciparum* malaria transmission in Ouélessébougou, Mali: a five-arm, phase 2, single-blind, randomised controlled trial

**DOI:** 10.1016/j.lanmic.2024.100966

**Published:** 2025-02

**Authors:** Almahamoudou Mahamar, Leen N Vanheer, Merel J Smit, Koualy Sanogo, Youssouf Sinaba, Sidi M Niambele, Makonon Diallo, Oumar M Dicko, Richard S Diarra, Seydina O Maguiraga, Ahamadou Youssouf, Adama Sacko, Sekouba Keita, Siaka Samake, Adama Dembele, Karina Teelen, Yahia Dicko, Sekou F Traore, Arjen Dondorp, Chris Drakeley, William Stone, Alassane Dicko

**Affiliations:** a Malaria Research and Training Centre, Faculty of Pharmacy and Faculty of Medicine and Dentistry, University of Sciences, Techniques and Technologies of Bamako, Bamako, Mali; b Department of Infection Biology, London School of Hygiene & Tropical Medicine, London, UK; c Department of Medical Microbiology and Radboud Center for Infectious Diseases, Radboud University Medical Center, Nijmegen, Netherlands; d Mahidol-Oxford Tropical Medicine Research Unit, Faculty of Tropical Medicine, Mahidol University, Bangkok, Thailand; e Centre for Tropical Medicine and Global Health, Nuffield Department of Clinical Medicine, University of Oxford, Oxford, UK

## Abstract

**Background:**

Triple artemisinin-based combination therapies (TACTs) can delay the spread of antimalarial drug resistance. Artesunate–amodiaquine is widely used for uncomplicated *Plasmodium falciparum* malaria. We therefore aimed to determine the safety and efficacy of artemether–lumefantrine–amodiaquine and artesunate–amodiaquine with and without single low-dose primaquine for reducing gametocyte carriage and transmission to mosquitoes.

**Methods:**

We did a five-arm, single-blind, phase 2 randomised controlled trial at the Ouélessébougou Clinical Research Unit of the Malaria Research and Training Centre of the University of Sciences, Techniques and Technologies of Bamako in Mali. Eligible participants were aged 10–50 years, with asymptomatic *P falciparum* microscopy-detected gametocyte carriage. Eligible participants were randomly allocated (1:1:1:1:1) to receive either artemether–lumefantrine, artemether–lumefantrine–amodiaquine, artemether–lumefantrine–amodiaquine plus primaquine, artesunate–amodiaquine, or artesunate–amodiaquine plus primaquine. Treatment regimens were administered on days 0, 1, and 2; primaquine was given as a single dose on day 0. All staff except the trial pharmacist and participants were masked to the treatment allocation. The primary outcome was the median percentage change in mosquito infection rate between pretreatment and 2 days after treatment initiation, assessed by direct membrane feeding assay. Data were analysed using a per-protocol analysis. This study is registered with ClinicalTrials.gov, NCT05550909.

**Findings:**

Between Oct 16, 2022, and Dec 28, 2022, a total of 1249 individuals were screened; of whom, 100 were enrolled and randomly assigned to one of the five treatment groups (20 per group). Before treatment, 61 (61%) of 100 participants were infectious to mosquitoes, with a median of 7·3% (IQR 3·2 to 23·5) of mosquitoes becoming infected. Among infectious participants, the median percentage reduction in mosquito infection rate between pretreatment and 2 days after treatment was 100% (IQR 100 to 100) in the artemether–lumefantrine (p=0·0018), artemether–lumefantrine–amodiaquine (p=0·0018), and artemether–lumefantrine–amodiaquine plus primaquine (p=0·0009) treatment groups. In the artesunate–amodiaquine group the median percentage reduction in mosquito infection rate was only 32% (IQR –10·9 to 79·4; p=0·19), whereas a 100% median reduction was seen in the artesunate–amodiaquine plus primaquine group (IQR 100 to 100; p=0·0009). At day 2, two (10%) of 20 participants in the artemether–lumefantrine group, two (11%) of 19 in the artemether–lumefantrine–amodiaquine group, and 15 (75%) of 20 in the artesunate–amodiaquine group infected any number of mosquitoes whereas no infected mosquitoes were observed at this timepoint in the groups with primaquine. 85 (85%) of 100 participants had a total of 262 adverse events during follow-up; of which, 181 (69%) were categorised as mild and 81 (31%) as moderate. No serious adverse events were reported.

**Interpretation:**

Our findings support the effectiveness of artemether–lumefantrine alone or as part of TACT for preventing nearly all human–mosquito malaria parasite transmission within 48 h. By contrast, substantial transmission was observed following treatment with artesunate–amodiaquine. The addition of a single low dose of primaquine blocks transmission to mosquitoes rapidly regardless of schizonticide.

**Funding:**

Bill & Melinda Gates Foundation.

## Introduction

Malaria morbidity and mortality remains unacceptably high.^1^ The emergence and spread of partial resistance against artemisinin derivatives, the main component of artemisinin-based combination therapies (ACTs), in southeast Asia^2^^,^^3^ and east Africa^4^^,^^5^ is threatening to increase malaria cases and deaths. Antimalarial treatments designed to slow the spread of resistance are therefore needed, either through novel combinations of existing drugs or supplementation with drugs that have specific effects on gametocytes, the sexual life stages responsible for maintaining parasite transmission. For optimal use, the understanding of how effective current and future antimalarials combat gametocytes is essential, and how this effect translates into reductions in transmission to mosquitoes.

**Research in context**

**Evidence before this study**

We searched PubMed on Nov 13, 2023, with no publication date or language restrictions, for studies assessing the post-treatment transmission of artemether–lumefantrine–amodiaquine with the following search terms: “([Artemether-lumefantrine] OR [Coartem] OR [Riamet])” AND “([Amodiaquine] OR [Flavoquine] OR [Triple ACT] OR [TACT] OR [Triple Artemisinin-based Combination therapy] OR [Triple therapy])” AND “([Plasmodium falciparum])” AND “([Gametocytocidal] OR [Gametocytes])” AND “([Transmission])”. The following search terms were added to search for studies using artemether–lumefantrine–amodiaquine plus primaquine: AND “([Primaquine] OR [Jasoprim] OR [Malirid] OR [Neo-Quipenyl] OR [Pimaquin] OR [Primachina] OR [Primacin] OR [Primaquina] OR [Remaquin])”. A second search was done for studies assessing the post-treatment transmission of artesunate–amodiaquine, with the following search terms: “([Artesunate-amodiaquine] OR [Camoquin] OR [Coarsucam] OR [Artesunat Plus] OR [TesquinCare])” AND “([Plasmodium falciparum])” AND “([Gametocytocidal] OR [Gametocytes])” AND “([Transmission])”. The aforementioned search terms for primaquine were added to search for studies using artesunate–amodiaquine plus primaquine.

The initial search (without the primaquine search terms) yielded nine studies: seven did not assess the combination of artemether–lumefantrine–amodiaquine and two were non-clinical trials. After addition of primaquine search terms, only one study was found, which assessed the effectiveness and post-treatment gametocyte density of four artemisinin-based combination therapies (ACTs) with or without primaquine; however, the combination of artemether–lumefantrine–amodiaquine was not tested. The second search found 26 studies: seven assessed safety and gametocyte carriage after artesunate–amodiaquine treatment but did not include mosquito feeding assays, 15 only tested artesunate and amodiaquine separately or in combination with other drugs, and four were non-clinical trials. The duration of gametocyte carriage, determined by microscopy, observed in these seven trials after treatment with artesunate–amodiaquine ranged from 21 days to persisting past 28 days after treatment initiation. Narrowing this search to include studies assessing artesunate–amodiaquine in combination with primaquine identified only two relevant trials, both of which assessed safety and efficacy against gametocytes determined by microscopy, but neither performed mosquito feeding assays. Both studies found that after artesunate–amodiaquine plus primaquine treatment gametocyte densities decreased to zero within 21–28 days.

**Added value of this study**

Our study provides valuable data for the extent of transmission after artemether–lumefantrine, artemether–lumefantrine–amodiaquine, and artesunate–amodiaquine in a highly infectious population sample. This study is the first assessment of artemether–lumefantrine–amodiaquine with and without single low-dose primaquine on gametocyte densities and transmission, using mosquito feeding assays. We show that artemether–lumefantrine–amodiaquine has potent transmission-blocking activity, even in the absence of primaquine. However, gametocyte densities declined more rapidly when primaquine was added and this combination blocks transmission even after gametocyte enrichment of the mosquito blood meal. We also provide the first evidence of continued transmission up until day 28 after artesunate–amodiaquine. Notably, we provide the first data demonstrating that the addition of a single-low dose of primaquine to artesunate–amodiaquine completely annuls transmission by day 2 after treatment. Lastly, we concentrated gametocytes in the mosquito blood meal to gain insights into the transmission-blocking mechanisms of antimalarial drugs.

**Implications of all the available evidence**

Our study findings are consistent with previous evidence that artemether–lumefantrine has potent transmission-blocking activity. We found that the addition of amodiaquine to artemether–lumefantrine did not influence gametocyte densities or transmission. However, the addition of a single-low dose of primaquine to the triple ACTs achieved a near-complete clearance of gametocytes by day 7, which is a more rapid clearance than previously observed after artemether–lumefantrine plus primaquine. In line with this finding, gametocyte enrichment enhanced transmission in the artemether–lumefantrine and artemether–lumefantrine–amodiaquine groups at day 2, but not in the group with added primaquine. This finding provides evidence that all gametocytes and transmission are completely annulled post-artemether–lumefantrine–amodiaquine plus primaquine treatment.

In addition, this study’s findings contribute to the expanding body of research supporting the incorporation of a single low-dose primaquine regimen with ACT as an immediate measure to halt the further transmission of *Plasmodium falciparum* malaria. The available data for the effects of artesunate–amodiaquine alone provide a resource to policy makers considering treatment options, and our findings on the efficacy of artesunate–amodiaquine with a single-low does primaquine support WHO’s recommendation of combining ACTs with single low-dose primaquine to prevent transmission in areas aiming to eliminate malaria or fighting the spread of antimalarial drug resistance.

Triple ACTs (TACTs) combine an existing ACT with a second partner drug that is slowly eliminated, to reduce the likelihood of incomplete parasite clearance and thus delay the spread of artemisinin resistance.^6^ Artemether–lumefantrine–amodiaquine is a TACT that has been proven safe, well tolerated, and efficacious for the treatment of uncomplicated *Plasmodium falciparum* malaria, including in areas with artemisinin and partner drug resistance.^7^^,^^8^ The effect of artemether–lumefantrine–amodiaquine on mature gametocytes and infectivity is unknown. Artesunate–amodiaquine is the first-line ACT for uncomplicated *P falciparum* malaria in many countries,^9^ but its efficacy in reducing transmission has not been directly tested. Studies assessing gametocyte carriage after artesunate–amodiaquine reported persistent gametocyte carriage after treatment for 21 days or more, but without transmission assays the infectivity of these persisting gametocytes cannot be confirmed.^10^, ^11^, ^12^

Although artemisinin-based treatments have superior gametocytocidal properties to non-artemisinin-based treatments,^13^ with artemether–lumefantrine being the most potent,^14^ the transmission-reducing activities of ACTs vary widely.^14^, ^15^, ^16^ By contrast, the 8-aminoquinoline primaquine is a potent gametocytocidal drug that, at a single low-dose (0·25 mg/kg), blocks transmission within 48 h of treatment. Since 2015, WHO recommends the addition of a single low-dose of primaquine to ACTs to reduce *P falciparum* transmission.^17^ The gametocytocidal and transmission-reducing activities of single low-dose primaquine have been assessed in combination with dihydroartemisinin–piperaquine, pyronaridine–artesunate, and artemether–lumefantrine,^14^, ^15^, ^16^^,^^18^^,^^19^; however, combining artemether–lumefantrine–amodiaquine or artesunate–amodiaquine with a single low-dose primaquine for *P falciparum* transmission reduction has not yet been tested.

In this study, we aimed to determine the safety and efficacy of artemether–lumefantrine–amodiaquine and artesunate–amodiaquine with and without single low-dose primaquine for reducing the transmission of *P falciparum* gametocytes.

## Methods

### Study design and participants

We did a five-arm, single-blind, phase 2 randomised controlled trial at the Ouélessébougou Clinical Research Unit of the Malaria Research and Training Centre (MRTC) of the University of Sciences, Techniques and Technologies of Bamako in Mali. Ouélessébougou is a commune that includes the town of Ouélessébougou and 44 surrounding villages, which have a total of approximately 50 000 inhabitants. Malaria transmission is highly seasonal, tied to the rainy season occurring from July to November. In children older than 5 years, the prevalence of *P falciparum* malaria varies between 50% and 60% and the prevalence of gametocytes varies between 20% and 25% during the transmission season. 2 days before the start of enrolment, the study team met with community leaders, village health workers, and heads of households from each village, before the commencement of screening, to explain the study and obtain verbal assent to undertake screening. Village health workers subsequently used a door-to-door approach to inform all available households of the date and location where consenting and screening would take place.

We included participants in the trial if they met the following criteria: positive for *P falciparum* gametocytes by microscopy (ie, ≥1 gametocytes observed in a thick film against 500 white blood cells, equating to 16 gametocytes per μL with a standard conversion of 8000 white blood cells per μL); absence of other non-*P falciparum* species on the blood film; haemoglobin density of 10 g/dL or more; aged between 10 and 50 years; bodyweight of 80 kg or less; no clinical signs of malaria, defined by fever (≥37·5°C); and no signs of acute, severe, or chronic disease. The exclusion criteria included pregnancy (tested at enrolment by urine test) or lactation, allergies to any of the study drugs, use of other medication (except for paracetamol or aspirin, or both), use of antimalarial drugs over the past week, history of prolongation of the corrected QT interval, documented or self-reported history of cardiac conduction problems or epileptic seizures, and blood transfusion in the last 90 days. A full list of the exclusion criteria is detailed in appendix 2 (p 11). We chose to recruit only asymptomatic individuals to increase the likelihood of observing high gametocyte densities.^20^

Before screening and study enrolment, participants provided written informed consent (≥18 years) or written parental consent (10–17 years). In addition to parental consent, oral assent was sought for individuals aged 10–17 years. Ethical approval was granted by the Ethics Committee of the University of Sciences, Techniques and Technologies of Bamako (Bamako, Mali; 2022/244/CE/USTTB), and the Research Ethics Committee of the London School of Hygiene & Tropical Medicine (London, UK; 28014). The study protocol is provided in appendix 2.

### Randomisation and masking

Participants were randomly allocated (1:1:1:1:1) to five treatment groups: artemether–lumefantrine; artemether–lumefantrine–amodiaquine; artemether–lumefantrine–amodiaquine plus primaquine; artesunate–amodiaquine; and artesunate–amodiaquine plus primaquine. An independent MRTC statistician randomly generated the treatment assignment using Stata (version 16), which was linked to participant identification numbers. The statistician prepared sealed, opaque envelopes with the participant identification number on the outside and treatment assignment inside, which were sent to the MRTC study pharmacist. Study participants were aware of the allocated treatment. The study pharmacist provided treatment and was consequently not masked to treatment assignment; staff involved in assessing safety, infectivity, and laboratory outcomes were masked.

### Procedures

Artesunate–amodiaquine and artemether–lumefantrine treatment (Guilin Pharmaceutical, Shanghai, China) was administered over 3 days (days 0, 1, and 2) as per manufacturer instructions. Participants in the artemether–lumefantrine–amodiaquine groups were treated with standard doses (Guilin Pharmaceutical, Shanghai, China) over 3 days (days 0, 1, and 2) as per manufacturer instructions. A single dose of 0·25 mg/kg primaquine (ACE Pharmaceuticals, Zeewolde, Netherlands) was administered on day 0 in parallel with the first dose of ACT or TACT, as described previously.^19^ Details about the dosing of these antimalarials are shown in appendix 1 (p 2).

Participants received a full clinical and parasitological examination on days 2, 7, 14, 21, and 28 after receiving the first dose of the study drugs (appendix 1 p 3). Giemsa-stained thick film microscopy was performed as described previously,^19^ with gametocytes counted against 500 white blood cells and asexual stages counted against 200 white blood cells. Total nucleic acids were extracted for molecular gametocyte quantification using a MagNAPure LC automated extractor (Total Nucleic Acid Isolation Kit-High Performance; Roche Applied Science, Indianapolis, IN, USA). Female and male gametocytes were quantified in a multiplex reverse transcriptase quantitative PCR (RT-qPCR) assay (appendix 1 p 4).^21^ Samples were classified as negative for a particular gametocyte sex if the RT-qPCR quantified density of gametocytes of that sex was less than 0·01 gametocytes per μL (ie, one gametocyte per 100 μL of blood sample). Haemoglobin density (g/dL) was measured from fingerprick samples using a haemoglobin analyser (HemoCue; AB Leo Diagnostics, Helsingborg, Sweden) or using an automatic haematology analyser (HumaCount 5D; Human Diagnostics Worldwide, Wiesbaden, Germany) from venous blood samples. Additional venous blood samples were taken for biochemical and infectivity assessments on days 0, 2, 7, and 14 in all treatment groups (appendix 1 p 3). Concentrations of aspartate aminotransferase, alanine aminotransferase, and blood creatinine were measured using the automatic biochemistry analyser Human 100 (Human Diagnostics Worldwide, Wiesbaden, Germany). For each assessment of infectivity, about 75 locally insectary-reared female *Anopheles gambiae (s.l.)* mosquitoes were allowed to feed for 15–20 min on venous blood samples (Lithium Heparin VACUETTE tube; Greiner Bio-One, Kremsmünster, Austria) through a prewarmed glass membrane feeder system (Coelen Glastechniek, Weldaad, Netherlands). Mosquitoes that had taken no bloodmeal or a partial bloodmeal were discarded; surviving blood-fed mosquitoes were dissected on the seventh day after feeding. Midguts were stained with 1% mercurochrome and examined for the presence and density of oocysts by one expert microscopist; positive midguts were confirmed by a second expert microscopist.

To investigate whether early post-treatment transmission-blocking was due either to insufficient gametocyte densities or drug-induced sterilisation effects, a separate venous blood sample (from baseline [day 0] and day 2 only) was processed by magnetic-activated cell sorting to enrich its gametocyte content before mosquito feeding in transmission assays. Gametocytes in the infected whole blood sample were concentrated by magnetic-activated cell sorting using a QuadroMACS separator and magnetic-activated cell sorting LS columns (MiltenyiBiotech, Bisley, UK) as previously described.^22^ Briefly, MACS LS columns were equilibrated with 1 mL of warm incomplete medium, followed by 3 mL of infected whole blood and 2 mL medium wash. LS columns were then removed from the magnet, and gametocytes were eluted in 4 mL of warm medium. Flow-through and gametocyte fractions were then centrifuged (2000 rotations per min for 5 min at 37°C). The medium was carefully removed, and the gametocyte pellet was resuspended in 450 μL warm malaria naive serum and 600 μL of the same participants packed cells. The entire magnetic-activated cell sorting procedure was done in a 37°C cabinet incubator.

### Outcomes

The primary outcome was median percentage change in mosquito infection rate between pretreatment and 2 days after treatment initiation. Secondary outcomes were mosquito infection metrics (infectious participants [ie, infected any number of mosquitoes], mosquito infection rate, and oocyst density) at prespecified timepoints (days 0, 2, 7, 14, 21, and 28); gametocyte and asexual parasite prevalence, density, gametocyte circulation time, area under the curve (AUC) of gametocyte density over time, and sex ratio (ie, proportion of gametocytes that were male or female); and safety assessments including incidence of clinical and laboratory adverse events. Differences in all transmission metrics, gametocyte, asexual stages, and safety outcomes were compared between matched treatment groups (ie, artemether–lumefantrine *vs* artemether–lumefantrine–amodiaquine and artemether–lumefantrine–amodiaquine plus primaquine, artesunate–amodiaquine *vs* artesunate–amodiaquine plus primaquine) as secondary outcomes.

Primary and secondary analyses of mosquito infection rate and oocyst density metrics were performed on participants infectious at baseline, but are shown for all participants in appendix 1 (p 6). Exploratory outcomes included mosquito infection metrics after gametocyte enrichment, for within and between treatment group comparison. Gametocyte infectivity was assessed as an exploratory outcome using logistic regression models adjusted for gametocyte density, wherein the shape of the relationship between gametocyte density and mosquito infection rate was estimated using fractional polynomials.

Adverse events were graded by the study clinician for severity (mild, moderate, or severe) and relatedness to study medication (unrelated or unlikely, possibly, probably, or definitely related). A reduction in haemoglobin concentration of 40% or more from baseline was categorised as a haematological severe adverse event. An external data safety and monitoring committee was assembled before the trial. Safety data were discussed after enrolment of 50 participants, and after the final follow-up visit of the last participant.

### Statistical analysis

Sample size was informed by previous trials in the study setting using a mixed effects logistic regression model that accounted for correlation between mosquito observations from the same participant^14^, ^15^, ^16^^,^^18^^,^^19^ and expecting a reduction in infectivity of 90% as previously detected for a single low-dose of primaquine.^15^^,^^19^ When including 20 participants per group and dissecting 50 mosquitoes per participant per timepoint, we calculated 92% empirical power to detect more than 85% reduction in infectivity with a one-tailed test with an α of 0·05 level of significance. The sample size was not designed for between-group comparisons and any comparison of transmission-blocking effects between groups is secondary and limited to matched treatment groups. Mosquito infectivity was assessed at three levels: the percentage of participants infectious to any number of mosquitoes (ie, infectious participants), the proportion of mosquitoes infected with any number of oocysts (ie, mosquito infection rate), and the mean number of oocysts in a sample of mosquitoes (ie, oocyst density).

The proportion of infectious participants and the prevalence of gametocytes and asexual stage parasites were compared between treatment groups using one-sided Fisher’s exact tests and within groups using McNemar tests. Mosquito infection rate was compared within groups (relative to baseline) by Wilcoxon sign rank test (*Z* score) and between groups by linear regression adjusted for baseline mosquito infection rate (*t* score, coefficient with 95% CI). For all direct membrane feeding assays (before and after gametocyte enrichment), the proportion of infectious participants was compared between groups (direct membrane feeds before gametocyte concentration as reference) and within groups (relative to baseline) using one-sided Fisher’s exact tests. Haemoglobin concentrations were compared using paired *t* tests (*t* score) for within-group analyses and linear regression adjusted for baseline levels of each measure for between-group analyses (*t* score, coefficient with 95% CI). Percentage change from baseline was analysed using two-sample *t* tests for between-group analysis and paired *t* tests (*t* score) for within-group analysis.

The proportion of gametocytes that were male was analysed for all values with total gametocyte densities of 0·2 gametocytes per μL or more, ensuring accurate quantification of sex ratios. Gametocyte circulation time was calculated to determine the mean number of days that a mature gametocyte circulates in the blood before clearance, using a deterministic compartmental model that assumes a constant rate of clearance and has a random effect to account for repeated measures on participants, as described previously.^23^ Differences in circulation time between groups and between gametocyte sexes were estimated in the model. Statistical theory shows that these parameter estimates follow a t-distribution. AUC of gametocyte density per participant over time was calculated using the linear trapezoid method and was analysed by fitting linear regression models to the log_10_ adjusted AUC values, with adjustment for baseline gametocyte density (*t* score, coefficient with 95% CI). All other analyses of quantitative data were done using Wilcoxon sign rank tests (*Z* score) and Wilcoxon rank-sum tests (*Z* score). All comparisons were defined before study completion and analyses were not adjusted for multiple comparisons. For all analyses, the threshold for statistical significance was set at p<0·05.

Statistical analysis was conducted using Stata (version 17.0) and SAS (version 9.4). Data visualisation was performed using the R-based *ggplot2* package (version 4.3.2) and Stata-based graphics (version 17.0). This trial is registered with ClinicalTrials.gov, number NCT05550909.

### Role of the funding source

The funder of the study had no role in study design, data collection, data analysis, data interpretation, or writing of the report.

## Results

**Figure 1:**
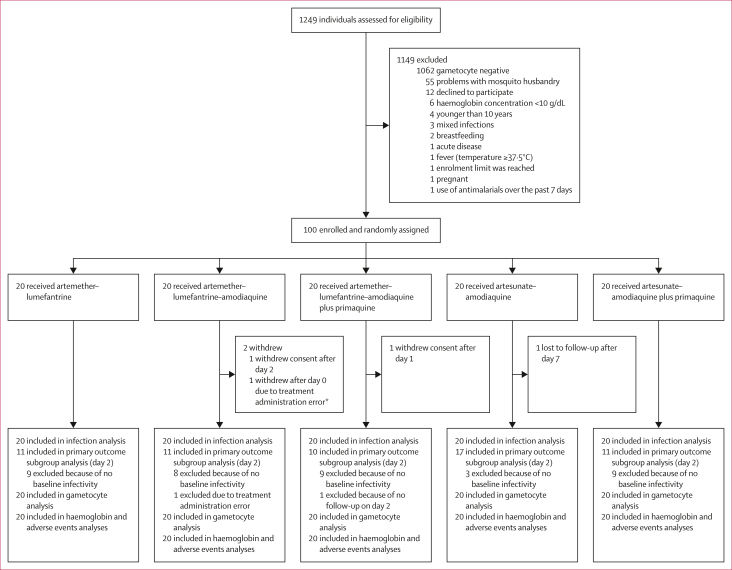
Trial profile 96 (96%) of 100 participants completed all visits to day 28 (two [10%] of 20 in the artemether–lumefantrine–amodiaquine group, one [5%] in the artemether–lumefantrine–amodiaquine plus primaquine group, and one [5%] in the artesunate–amodiaquine plus primaquine group did not complete all visits). ∗One participant randomly allocated to the artemether–lumefantrine–amodiaquine group was given erroneous treatment on day 1. All measures following this error were removed from analysis.

Between Oct 16, 2022, and Dec 28, 2022, a total of 1249 eligible individuals were screened; of whom, 100 were enrolled and randomly assigned to one of the five treatment groups (20 participants per group; figure 1). Participant characteristics were similar between the treatment groups, although the proportion of infectious participants at baseline was higher in the artesunate–amodiaquine group (tables 1, 2). The primary outcome was recorded on day 2 of follow-up, with 98 (98%) of 100 participants completing this study visit (one in the artemether–lumefantrine–amodiaquine group and one in the artemether–lumefantrine–amodiaquine plus primaquine group did not complete this visit). 96 (96%) of 100 participants completed all visits to day 28 (two in the artemether–lumefantrine–amodiaquine, one in the artemether–lumefantrine–amodiaquine plus primaquine, and one in the artesunate–amodiaquine group did not complete all visits).

**Table 1:** Baseline characteristics before treatment

	Artemether–lumefantrine (n=20)	Artemether–lumefantrine–amodiaquine (n=20)	Artemether–lumefantrine–amodiaquine plus primaquine (n=20)	Artesunate–amodiaquine (n=20)	Artesunate–amodiaquine plus primaquine (n=20)
Age (years)	13·0 (11·0–18·5)	13·0 (11·5–28·0)	13·0 (11·0–15·0)	12·0 (10·0–16·0)	12·5 (11·5–20·0)
Sex					
Female	11 (55%)	10 (50%)	12 (60%)	11 (55%)	7 (35%)
Male	9 (45%)	10 (50%)	8 (40%)	9 (45%)	13 (65%)
Haemoglobin (g/dL)	11·8 (11·5–13·8)	12·1 (11·3–12·6)	11·8 (11·1–12·1)	11·4 (10·9–12·3)	11·7 (11·2–12·8)
Gametocyte prevalence	20 (100%)	20 (100%)	20 (100%)	20 (100%)	20 (100%)
Gametocyte density (parasites per μL)	31·0 (19·9–92·7)	28·6 (11·5–130·5)	42·3 (11·8–97·0)	52·5 (33·6–129·0)	24·8 (10·7–115·2)
Asexual parasite prevalence	10 (50%)	6 (30%)	5 (25%)	8 (40%)	8 (40%)
Asexual parasite density (parasites per μL)	37·9 (0·0–300·0)	0·0 (0·0–79·8)	0·0 (0·0–37·6)	0·0 (0·0–1654·9)	0·0 (0·0–720·0)

Data are median (IQR) or n (%).

**Table 2:** Median percentage reduction in mosquito infection rate for participants infectious before treatment

	Infectious participants	Median mosquito infection rate	Median percentage reduction in mosquito infection rate	p value∗	p value†
**Baseline**					
Artemether–lumefantrine	11/20 (55%)	4·5% (3·3–44·1)	··	Ref	··
Artemether–lumefantrine–amodiaquine	11/20 (55%)	10·9% (3·3–32·3)	··	Ref	··
Artemether–lumefantrine–amodiaquine plus primaquine	11/20 (55%)	4·1% (2·1–8·8)	··	Ref	··
Artesunate–amodiaquine	17/20 (85%)	7·3% (1·9–23·5)	··	Ref	··
Artesunate–amodiaquine plus primaquine	11/20 (55%)	9·3% (1·8–36·2)	··	Ref	··
**Day 2**					
Artemether–lumefantrine	2/20 (10%)	0% (0–0)	100% (100 to 100)	0·0018	Ref
Artemether–lumefantrine–amodiaquine	2/19 (11%)	0% (0–0)	100% (100 to 100)	0·0018	1·0000
Artemether–lumefantrine–amodiaquine plus primaquine	0/19	0% (0–0)	100% (100 to 100)	0·0009	0·15
Artesunate–amodiaquine	15/20 (75%)	5% (1·5–9·7)	31·7% (–10·87 to 79·39)	0·19	Ref
Artesunate–amodiaquine plus primaquine	0/20	0% (0–0)	100% (100 to 100)	0·0009	0·0001

Data are n/N (%) or median (IQR), unless otherwise specified. Participants were classed as infectious if direct membrane feeding assays resulted in at least one mosquito with any number of oocysts. All values are for participants who were infectious to mosquitoes before treatment. The range of median percentage reduction in mosquito infection rate between pretreatment and 2 days after treatment was 83·1 to 100 in the artemether–lumefantrine group, 82·4 to 100 in the artemether–lumefantrine–amodiaquine group, 100 to 100 in the artemether–lumefantrine–amodiaquine plus primaquine group, –112·6 to 100 in the artesunate–amodiaquine group, and 100 to 100 in the artesunate–amodiaquine plus primaquine group.

∗ Within-group comparison of median reduction in mosquito infection rate by Wilcoxon signed rank test (day 0 as reference, primary outcome).

† Between-group comparison of median reduction in mosquito infection rate (ie, artemether–lumefantrine *vs* artemether–lumefantrine–amodiaquine and artemether–lumefantrine–amodiaquine plus primaquine, artesunate–amodiaquine *vs* artesunate–amodiaquine plus primaquine) by Wilcoxon rank-sum test. Full details about mosquito feeding assay outcomes are summarised in appendix 1 (p 5). Ref=reference.

**Figure 2:**
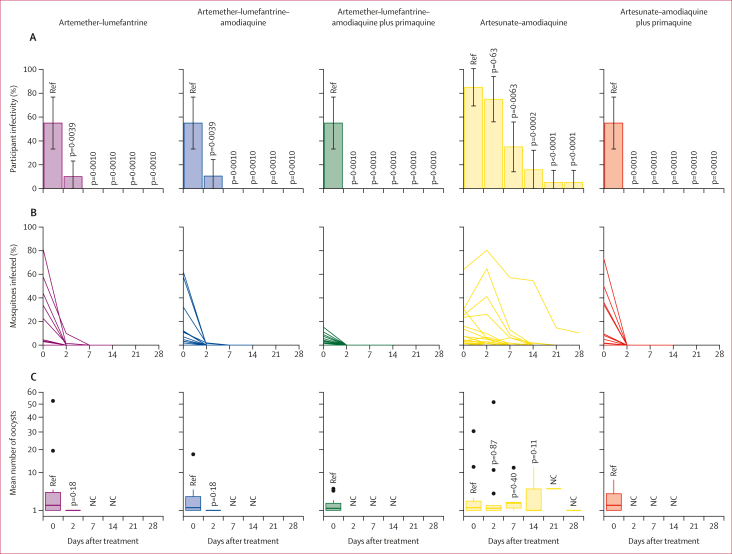
Participant infectivity and proportion of mosquitoes infected in direct membrane feeding assays (A) The proportion of infectious individuals was compared within treatment groups using McNemar tests. The denominator for participant infectivity is the total number of participants still enrolled at the given timepoint, rather than the number tested for infectivity at that timepoint. Infectivity assays were discontinued after 14 days when a participant did not infect any mosquitoes at two subsequent timepoints and were thereafter considered non-infectious. Mosquito feeding assays at days 21 and 28 were only done in the artesunate–amodiaquine group (seven [37%] of 19 at day 21 and three [16%] of 19 at day 28). Error bars are 95% CIs. (B) Each line represents one participant. Statistical analyses are summarised in appendix 1 (p 5). (C) Box plots show the median (central line), IQR (box limits), upper and lower quartiles plus 1·5 IQR (whiskers), and outliers for mean oocyst densities in infected mosquitoes within each participant. The mean number of oocysts was compared to baseline within treatment groups using the Wilcoxon sign-rank test. NC=not calculable. Ref=reference.

The median number of mosquitoes dissected in an individual mosquito feeding experiment was 60 (IQR 54 to 64). Before treatment, 61 (61%) of 100 participants were infectious to mosquitoes (17 participants randomly allocated to the artesunate–amodiaquine group and 11 in all other treatment groups), with a median of 7·3% (IQR 3·2 to 23·5) of mosquitoes becoming infected. The median number of oocysts per infected mosquito was 1·3 (IQR 1·0 to 2·7). At day 2 a significant within-person reduction in mosquito infection rate relative to baseline was observed in all groups except for the artesunate–amodiaquine group, with a median percentage reduction in mosquito infection rate of 100% (IQR 100 to 100) in the artemether–lumefantrine (p=0·0018), artemether–lumefantrine–amodiaquine (p=0·0018), and artemether–lumefantrine–amodiaquine plus primaquine (p=0·0009) treatment groups. In the artesunate–amodiaquine group the median percentage reduction in mosquito infection rate was only 32% (IQR –10·9 to 79.4; p=0·19), whereas a 100% median reduction was seen in the artesunate–amodiaquine plus primaquine group (IQR 100 to 100; p=0·0009). At day 2, two (10%) of 20 participants in the artemether–lumefantrine group, two (11%) of 19 in the artemether–lumefantrine–amodiaquine group, and 15 (75%) of 20 in the artesunate–amodiaquine group infected any number of mosquitoes. No participants remained infectious to mosquitoes at day 2 in the treatment groups with primaquine (table 2). At all timepoints after day 2, infectious participants were only found in the artesunate–amodiaquine group; seven (35%) of 20 at day 7, three (16%) of 19 at day 14, and one (5%) of 19 at days 21 and 28 (figure 2; appendix 1 p 5). Mosquito infection data for all participants, regardless of baseline infectivity, are presented in the appendix 1 (p 6).

Gametocyte enrichment by magnetic-activated cell sorting was performed on 95 blood samples collected before treatment on day 0 (baseline) and 94 blood samples that were collected on the second day after treatment initiation. Overall, gametocyte enrichment increased mosquito infection rates by a mean of 7·3% (SD 16·3; appendix 1 p 7). Comparing direct membrane feeds before enrichment with those after, the percentage of infectious participants increased in the enrichment-boosted group in all treatment groups at baseline (appendix 1 p 7); whereas, at day 2, the percentage of infectious participants increased for all treatment groups except for artemether–lumefantrine–amodiaquine plus primaquine, in which all participants remained non-infectious. In the artesunate–amodiaquine plus primaquine group, two initially non-infectious participants transmitted to one and four mosquitoes following gametocyte enrichment.

Asexual parasite densities, measured by microscopy, decreased rapidly after treatment initiation, with only one (5%) of 20 participants in both the artemether–lumefantrine and artesunate–amodiaquine groups retaining asexual stages at day 2; whereas, in all other treatment groups, no asexual parasites were observed after treatment initiation (appendix 1 p 9). Gametocyte densities declined over time in all treatment groups, although much more rapidly in those who received primaquine, with median gametocyte densities of 13·3 gametocytes per μL (IQR 5·6–21·0), 6·9 (1·1–49·9), and 31·7 (7·3–61·6) at day 7 in the groups without primaquine compared with median densities of 0·0 (0·0–0·0) and 0·2 (0·0–0·9) in the groups with primaquine (appendix 1 pp 10–11). 18 (95%) of 19 participants treated with artesunate–amodiaquine were still gametocyte positive (>1 gametocyte per 100 μL) at the final day of follow-up (day 28), whereas 16 (80%) of 20 in the artemether–lumefantrine and 13 (72%) of 18 in the artemether–lumefantrine–amodiaquine groups remained gametocyte positive at the same timepoint. Only one (5%) of 19 participants in the artemether–lumefantrine–amodiaquine plus primaquine group and one (5%) of 20 in the artesunate–amodiaquine plus primaquine group had persisting gametocytes at day 28. Total gametocyte circulation time was estimated at 6·1 days (95% CI 5·4–6·9) in the artemether–lumefantrine group, 6·0 days (5·2–6·8) in the artemether–lumefantrine–amodiaquine group, and 2·6 (2·1–3·1) in the artemether–lumefantrine–amodiaquine plus primaquine group (appendix 1 p 12); the same measure was estimated at 7·9 days (95% CI 6·7–9·3) in the artesunate–amodiaquine group and 3·3 days (2·8–3·8) in the artesunate–amodiaquine plus primaquine group. Gametocyte sex ratios showed a male bias from day 2 after starting treatment in the artemether–lumefantrine, artemether–lumefantrine–amodiaquine, and artemether–lumefantrine–amodiaquine plus primaquine groups; and from day 7 in the artesunate–amodiaquine plus primaquine group, with significantly more males in this group than in the artesunate–amodiaquine alone group (median proportion of male gametocytes of 1·0 [IQR 0·9–1·0] *vs* 0·6 [0·4–0·7]; p=0·0002; appendix 1 pp 11, 13–14). Too few gametocytes persisted to make conclusions about absolute per-gametocyte infectivity (appendix 1 p 15).

A significant difference was observed in the within-group reduction in mean haemoglobin density in all treatment groups at day 2 compared with baseline; however, by day 7, the haemoglobin concentrations had normalised in all groups and were comparable to baseline (appendix 1 pp 16–17). The greatest reduction in mean haemoglobin density in any treatment group or timepoint was 5·6% (SD 2·0; 95% CI 3·6–7·5) in the artesunate–amodiaquine group at day 2. No significant decreases were observed in percentage change in haemoglobin compared with baseline between treatment groups at any timepoint. The greatest reduction in haemoglobin density in any participant was 25·2% (from 14·3 g/dL at baseline to 10·7 g/dL at day 21 in a participant in the artemether–lumefantrine group). The lowest observed haemoglobin density in any participant and timepoint was 9 g/dL at baseline in a participant in the artesunate–amodiaquine group. No severe laboratory abnormalities occurred; all possibly drug-related laboratory abnormalities normalised on the subsequent visit (appendix 1 p 18).

Overall, 85 (85%) of 100 participants had a total of 262 adverse events during follow-up; of which, 181 (69%) were categorised as mild and 81 (31%) as moderate (table 3; appendix 1 p 19). No severe adverse events or serious adverse events occurred during the trial. The most common treatment-related adverse event was mild or moderate headache, which occurred in 43 (43%) of 100 participants (six [14%] in the artemether–lumefantrine group, nine [21%] in the artemether–lumefantrine plus amodiaquine group, 11 [26%] in the artemether–lumefantrine–amodiaquine plus primaquine group, eight [19%] in the artesunate–amodiaquine group, and nine [21%] in the artesunate–amodiaquine plus primaquine group). No significant differences were observed between treatment groups in the proportion of participants who experienced any adverse event (p=0·61), mild treatment-related adverse events (p=0·18), or moderate treatment-related adverse events (p=0·055) at any study visit.

**Table 3:** Frequency of adverse events

	Total (n=100)	Artemether–lumefantrine (n=20)	Artemether–lumefantrine–amodiaquine (n=20)	Artemether–lumefantrine–amodiaquine plus primaquine (n=20)	Artesunate–amodiaquine (n=20)	Artesunate–amodiaquine plus primaquine (n=20)
**All adverse events**	85 (85%)	18 (90%)	15 (75%)	18 (90%)	16 (80%)	18 (90%)
p value	0·612∗	Ref	0·407†	1·000†	Ref	0·661†
**Mild related adverse events**	47 (47%)	12 (60%)	7 (35%)	8 (40%)	7 (35%)	13 (65%)
p value	0·178∗	Ref	0·205†	0·343†	Ref	0·113†
**Moderate related adverse events**	26 (26%)	2 (10%)	7 (35%)	7 (35%)	8 (40%)	2 (10%)
p value	0·055∗	Ref	0·065127†	0·127†	Ref	0·065†
**Severe related adverse events**	0	0	0	0	0	0
p value	NC	Ref	NC	NC	ref	NC

Data are n (%), unless otherwise specified. If there were multiple episodes of adverse events per participant, the highest grade and most likely related to treatment is summarised in this table.

∗ p values are from Fisher’s exact tests for differences in proportion of participants with an adverse event between all groups.

† p values are from Fisher’s exact tests for differences in proportion of participants with an adverse event between the artemether–lumefantrine–amodiaquine or artemether–lumefantrine–amodiaquine plus primaquine groups and the artemether–lumefantrine reference group and artesunate–amodiaquine plus primaquine group and the artesunate–amodiaquine reference group. Ref=reference group. NC=not calculable.

## Discussion

To our knowledge, this study is the first clinical trial designed to test the gametocytocidal and transmission-blocking properties of the ACT artemether–lumefantrine–amodiaquine with and without primaquine and of artesunate–amodiaquine with and without primaquine. Within 48 h of treatment, transmission was greatly reduced in the artemether–lumefantrine and artemether–lumefantrine–amodiaquine groups, and completely annulled in both treatment groups with primaquine. By contrast, transmission to mosquitoes continued in a minority of participants until day 28 after treatment with artesunate–amodiaquine alone.

Calls for malaria eradication and the emergence and spread of drug resistance have reinforced the need to assess the effects of antimalarial drugs on gametocytes and their infectiousness.^17^^,^^24^ The addition of a second partner drug to ACTs could substantially delay the emergence and spread of artemisinin resistance and treatment failure. Lumefantrine and amodiaquine provide mutual protection against resistance development, and deployment of the TACT artemether–lumefantrine–amodiaquine is expected to extend the viability of artemisinin derivatives and both partner drugs.^6^ This study was not designed to investigate the clinical efficacy of TACT, nor had the study site recorded any partial artemisinin resistance at the time of the study. We found that both treatments with artemether–lumefantrine alone and with amodiaquine greatly reduced transmission by day 2 after treatment. The addition of a single low-dose primaquine only marginally enhanced this transmission-blocking effect. Gametocyte densities minimally differed between artemether–lumefantrine and artemether–lumefantrine–amodiaquine, but we observed a near complete clearance of gametocytes by day 7 in the group with an added single low-dose of primaquine. These observations align with recent data indicating that artemether–lumefantrine has potent transmission-blocking effects.^14^

Artesunate–amodiaquine is the first-line treatment for uncomplicated *P falciparum* malaria in many countries, yet its effect on mature gametocytes and transmission is unclear. In line with previous studies,^10^^,^^11^ we found that gametocyte carriage persisted in all participants treated with artesunate–amodiaquine until the end of follow-up (day 28) and three (16%) of 19 participants (three [18%] of 17 participants infectious at baseline) were still infectious to mosquitoes 14 days after initiation of treatment, with one participant remaining infectious until the end of follow-up (day 28). Moreover, one (5%) of 20 participants was infectious on day 2 but not at baseline, and one (5%) participant was infectious on day 7, but not at baseline or day 2. As transmission to mosquitoes involves an inherent stochastic element, the observation of no infected mosquitoes at one timepoint cannot rule out low levels of infectivity. This pattern suggests a possible role for differences in the drug susceptibility or exposure at different gametocyte developmental stages—ie, immature gametocytes might be released from sequestration in the bone marrow or spleen after treatment. The addition of a single low-dose of primaquine resulted in an enhanced clearance of gametocytes and achieved in a near-total reduction of transmission potential within 48 h.

The exploratory assay of magnetic gametocyte enrichment showed that the percentage of infectious participants increased at day 2 after treatment start in all groups except for the artemether–lumefantrine–amodiaquine plus primaquine group, although this increase was non-significant. This finding suggests that in all ACT only groups, the initial lack of infectivity is due to low gametocyte densities or sex ratio distortion rather than the sterilisation of either gametocyte sex. In the primaquine groups, the enrichment results were contradictory: the addition of primaquine to artemether–lumefantrine–amodiaquine blocked all transmission at day 2 even after gametocyte enrichment, suggestive of primaquine sterilising gametocytes before reducing their numbers significantly.^25^ Conversely, in the artesunate–amodiaquine plus primaquine group, two participants who were not infectious in standard feeding assays became infectious after gametocyte concentration. Although these samples are small and other factors such as variations in primaquine concentration have not been measured, this finding might indicate varying primaquine efficacy with different artemisinin therapies. Of relevance is that in the process of gametocyte enrichment, human plasma is replaced by malaria-naive serum, thereby removing potentially transmission-modulating antibodies or drugs that might affect parasite development upon mosquito ingestion.

The emergence of transmission after treatment, which was observed in the artesunate–amodiaquine group in standard feeding assays, has been seen previously after artemisinin^14^ and non-artemisinin treatments.^14^^,^^26^ Taken together with the observation that two initially non-infectious participants became infectious after gametocyte enrichment in the artesunate–amodiaquine plus primaquine group on day 2, we hypothesise that artesunate–amodiaquine might have lower efficacy on or exposure to immature, developing gametocytes than artemether–lumefantrine, and that gametocytes released from sequestration after treatment would be unaffected by primaquine’s active metabolites, which only circulate for a few hours (median elimination half-life of 4·7 hours).^27^ In addition, although artemether–lumefantrine–amodiaquine without primaquine prevents nearly all mosquito infections within 48 h, gametocytes in *PfKelch13* mutant infections might preferentially survive artemisinin exposure and infect mosquitoes.^28^ Our data support the suggestion from WHO’s malaria policy and advisory group to expand the focus on reducing parasite transmission with a single low-dose of primaquine in areas where partial artemisinin resistance has been detected.^29^

Previous studies reported a higher frequency of side-effects with the combination of partner drugs lumefantrine and amodiaquine than with lumefantrine alone,^7^^,^^8^ including vomiting, nausea, vertigo, and mild bradycardia. We did not see an increase in vomiting or nausea, and only a slight increase in vertigo related to the drug treatment, from one adverse event in the artemether–lumefantrine group, to three and six adverse events in the artemether–lumefantrine–amodiaquine groups with and without primaquine, respectively. Overall, all drug regimens were well tolerated, and no instances of cardiac adverse events or severe side-effects were reported.

Our study had some limitations. For instance, we assessed many secondary outcomes, and their interpretations therefore require caution because of issues of multiple testing. In addition, we recruited participants who had high densities of gametocytes, consistent with previous studies with similar outcomes and at the same study site.^15^^,^^16^^,^^18^^,^^19^ This approach allowed us to collect robust data for post-treatment transmissibility but does not represent the average gametocyte-infected participant. Consequently, our estimates of persistence of transmissible gametocytes primarily demonstrate the effect of antimalarial drugs on the transmission potential stemming from a comparatively small subset of highly infectious participants; although these effects would be the most important group for the drug regimens to work in. Lastly, the public health significance of our study findings needs to be validated through community trials focused on transmission outcomes. Mass administrations of primaquine or other gametocytocidal compounds (eg, alongside seasonal malaria chemoprophylaxis) might be necessary to achieve reductions in transmission at the community level.^30^ Conversely, given the negligible cost of primaquine, absence of safety concerns, and no obvious alternative, a compelling argument exists to add primaquine to slow the transmission of drug-resistant parasites.

In conclusion, our findings show that artemether–lumefantrine–amodiaquine can prevent nearly all mosquito infections, but reveal considerable post-treatment transmission after artesunate–amodiaquine. The addition of a single low-dose of primaquine is a safe and effective addition to artemether–lumefantrine–amodiaquine and artesunate–amodiaquine for blocking *P falciparum* transmission. Enriching the gametocyte content of mosquito blood meals in transmission assays shows that viable male and female gametocytes can persist at densities too low to result in mosquito infection at physiological concentrations, after treatment in the artemether–lumefantrine, artemether–lumefantrine–amodiaquine, artesunate–amodiaquine and artesunate–amodiaquine plus primaquine groups, but not in the artemether–lumefantrine–amodiaquine plus primaquine group. This finding strengthens the argument for the addition of a single-low dose of primaquine to block the transmission of artemisinin resistant gametocytes.

## Data sharing

Anonymised data reported in the manuscript will be made available to investigators who provide a methodologically sound proposal to the corresponding author. The study protocol is available in appendix 2. The data from this trial are accessible on Clinical Epidemiology Database Resources.

## Declaration of interests

We declare no competing interests.
