# Supplementary material for: Artemether–lumefantrine–amodiaquine or artesunate–amodiaquine combined with single low-dose primaquine to reduce Plasmodium falciparum malaria transmission in Ouélessébougou, Mali: a five-arm, phase 2, single-blind, randomised controlled trial
Source: Lancet Microbe. 2025 Feb;6(2):None. doi: 10.1016/j.lanmic.2024.100966 (PMC11798902; doi:10.1016/j.lanmic.2024.100966)
Supplement: Supplementary appendix 1 [file mmc1.pdf]

THE LANCET
Microbe

Supplementary appendix 1

This appendix formed part of the original submission and has been peer reviewed. We post it as supplied by the authors.

Supplement to: Mahamar A, Vanheer LN, Smit MJ, et al. Artemether–lumefantrine–amodiaquine or artesunate–amodiaquine combined with single low-dose primaquine to reduce *Plasmodium falciparum* malaria transmission in Ouélessébougou, Mali: a five-arm, phase 2, single-blind, randomised controlled trial. *Lancet Microbe* 2025. https://doi.org/10.1016/j.lanmic.2024.100966

SUPPLEMENTARY APPENDIX

Supplementary Information 1. Antimalarial treatment dosing *2*

Supplementary Figure 1. Schematic representation of sample collection and analysis pipeline *3*

Supplementary Table 1. Primer sequences and qPCR conditions for PfMGET CCp4 assay *4*

Supplementary Table 2. Infectivity to mosquitoes for individuals infectious at baseline *5*

Supplementary Table 3. Infectivity to mosquitoes for all individuals *6*

Supplementary Figure 2. Difference in mosquito infection rate and person infectivity before and after gametocyte enrichment *7*

Supplementary Table 4. Participant infectivity after gametocyte enrichment. *8*

Supplementary Table 5. Asexual parasite density by microscopy *9*

Supplementary Figure 3. Gametocyte density and prevalence by gametocyte sex *10*

Supplementary Table 6. Total gametocyte density, prevalence and sex ratio *11*

Supplementary Table 7. Gametocyte circulation time and area under the curve *12*

Supplementary Table 8. Female (CCP4) and male (PfMGET) gametocyte density and prevalence *13*

Supplementary Figure 4. Proportion of gametocytes that were male *14*

Supplementary Table 9. Gametocyte infectivity *15*

Supplementary Table 10. Haemoglobin density *16*

Supplementary Figure 5. Absolute haemoglobin density *17*

Supplementary Table 11. Biochemistry *18*

Supplementary Table 12. All adverse events *19*

References *20*

Supplementary Information 1. Antimalarial treatment dosing

1. Artemether-Lumefantrine (AL)

Participants in the AL, AL-AQ or AL-AQ+PQ groups were treated with standard doses of AL (Guilin Pharmaceutical, Shanghai, China) from day 0-2. Tablets containing 20 mg artemether and 120 mg lumefantrine will be administered per manufacturer guidelines as shown below:

**Bodyweight (kg)**	**20/120 mg tablet**		
	**D0**	**D1**	**D2**
5 to < 15 kg	1 disp tab x 2	1 disp tab x 2	1 disp tab x 2
15 to < 25 kg	2 disp tab x 2	2 disp tab x 2	2 disp tab x 2
25 to < 35 kg	3 tab x 2	3 tab x 2	3 tab x 2
≥ 35 kg	4 tab x 2	4 tab x 2	4 tab x 2

2. Primaquine (PQ)

Participants in the AL-AQ+PQ and AS-AQ+PQ groups received PQ (ACE Pharmaceuticals, Zeewolde, The Netherlands) at a single low dose of 0.25mg/kg as is currently recommended by the World Health Organization. The single dose of PQ was given on day 0 together with the first dose of AL or ASAQ, administered in an aqueous solution, according to a standard operating procedure (SOP) provided Sanofi as previously done at the study site when PQ was combined with DP, PA or AL (1–3).

3. Amodiaquine (AQ)

Participants in the AL-AQ and AL-AQ+PQ groups were given AQ as tablets of 153 mg (Guilin Pharmaceutical, Shanghai, China). The weight-based treatment schedule as shown below aims for a dosage of approximately 10 mg (7.7-15.3mg)/kg/day, given once or twice daily (together with artemether–lumefantrine) for three days:

**Bodyweight (kg)**	**153 mg tablet**					
	**D0 (0hr)**	**D0 (8hr)**	**D1 (24hr)**	**D1 36hr)**	**D2 (48hr)**	**D2 (60hr)**
10 to 19.9	1 tab	0 tab	1 tab	0 tab	1 tab	0 tab
20 to 29.9	1 tab	1 tab	1 tab	1 tab	1 tab	1 tab
30 to 54.9	2 tab	1 tab	2 tab	1 tab	2 tab	1 tab
55 to 80	3 tab	2 tab	3 tab	2 tab	3 tab	2 tab

4. Artesunate-Amodiaquine (AS-AQ)

Participants in the AS-AQ and AS-AQ+PQ groups received fixed-dose combination tablets containing 50mg/135 mg or 100mg/270 mg of artesunate/amodiaquine (Guilin Pharmaceutical, Shanghai, China). Tablets were administered according to manufacturer guidelines, as shown below:

**Weight**	**Tablets**	**D0**	**D1**	**D2**
9 to < 18 kg	50 mg AS/135 mg AQ base	1 tab	1 tab	1 tab
18 to < 36 kg	100 mg AS/270 mg AQ base<br>blister pack of 3 tab	1 tab	1 tab	1 tab
≥ 36 kg	100 mg AS/270 mg AQ base<br>blister pack of 6 tab	2 tab	2 tab	2 tab

Supplementary Figure 1. Schematic representation of sample collection and analysis pipeline

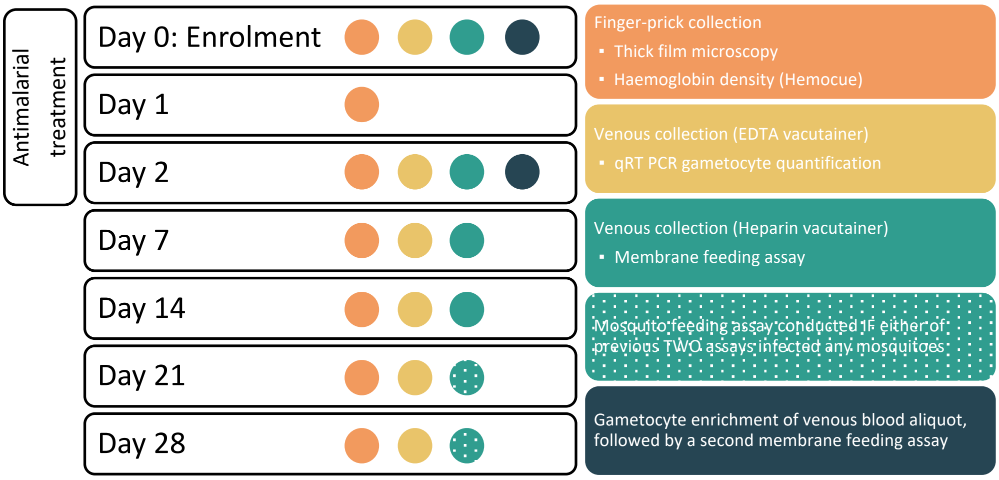

Supplementary Table 1. Primer sequences and qPCR conditions for PfMGET CCp4 assay

PfMGET Primer/Probe Sequences

Primers	Sequence
Primer-FW (5’-3’)	CGGTCCAAATATAAAAATCCTG
Primer-RV (5’-3’)	TGTG TAACG TATG ATTCATTTTC
Probe (5’-3’)	FAM-CAGCTCCAG CATTAAAACAC-BHQ1

CCp4 Primer/Probe Sequences

Primers	Sequence
Primer-FW (5’-3’)	CACATGAATATGAGAATAAAATTG
Primer-RV (5’-3’)	TAGGCGAACATGTGGAAAG
Probe (5’-3’)	TexasRed-AGCAACAACGGTATGTGCCTTAAAACG-BHQ2

Male and female gametocyte quantification was performed as described previously, using a multiplex RT-qPCR assay (4). Assays were run using commercial RT-qPCR mixes (Luna® Universal Probe One-Step RT-qPCR Kit, New England Biolabs, Ipswich, MA, USA). FW = Forward primer. RV = Reverse primer.

Supplementary Table 2. Infectivity to mosquitoes for individuals infectious at baseline

Day of follow-up	Treatment arm	Infectious individuals % (n/N) *	p-value¥	p-value†	Mosquito infection rate Median % (IQR) **	p-value¥	p-value†	Oocyst density Median (IQR) ***	p-value¥	p-value†
**Day 0**	**Overall**	61% (61/100)	·	·	7·3% (3·2-23.5)	·	·	1·3 (1-3·0)	·	·
	**AL**	55% (11/20)	*ref*	*ref*	4·5% (3·3-44·1)	*ref*	*ref*	1·7 (1-4·8)	*ref*	*ref*
	**AL-AQ**	55% (11/20)	*ref*	1·000	10·9% (3·3-32·3)	*ref*	0·646	1·3 (1-3·5)	*ref*	0·756
	**AL-AQ+PQ**	55% (11/20)	*ref*	1·000	4·1% (2·1-8·8)	*ref*	0·045	1·2 (1-2·5)	*ref*	0·535
	**AS-AQ**	85% (17/20)	*ref*	*ref*	7·3% (1·9-23·5)	*ref*	*ref*	1·3 (1-2·3)	*ref*	*ref*
	**AS-AQ+PQ**	55% (11/20)	*ref*	0·082	9·3% (1·8-36·2)	*ref*	0·370	1·7 (1-3·9)	*ref*	0·716
**Day 2**	**AL**	10% (2/20)	0·0039	*ref*	0% (0-0)	0·0033	*ref*	1·0 (1-1)	0·1797	*ref*
	**AL-AQ**	10·5% (2/19)	0·0039	0·678	0% (0-0)	0·0033	0·523	1·0 (1-1)	0·1797	*nc*
	**AL-AQ+PQ**	0% (0/19)	0·001	0·256	0% (0-0)	0·0033	0·788	*nc*	*nc*	*nc*
	**AS-AQ**	75% (15/20)	0·625	*ref*	5% (1·5-9·7)	0·6192	*ref*	1·2 (1-1·8)	0·8744	*ref*
	**AS-AQ+PQ**	0% (0/20)	0·001	<0·0001	0% (0-0)	0·0033	0·006	*nc*	*nc*	*nc*
**Day 7**	**AL**	0% (0/20)	0·001	*ref*	0% (0-0)	0·0033	*ref*	*nc*	*nc*	*ref*
	**AL-AQ**	0% (0/18)	0·001	*nc*	0% (0-0)	0·0033	*nc*	*nc*	*nc*	*nc*
	**AL-AQ+PQ**	0% (0/19)	0·001	*nc*	0% (0-0)	0·0033	*nc*	*nc*	*nc*	*nc*
	**AS-AQ**	35% (7/20)	0·0063	*ref*	0% (0-6·2)	0·001	*ref*	2·0 (1·6-2·2)	0·3991	*ref*
	**AS-AQ+PQ**	0% (0/20)	0·001	0·004	0% (0-0)	0·0033	0·045	*nc*	*nc*	*nc*
**Day 14**	**AL**	0% (0/20)	0·001	*ref*	0% (0-0)	0·0033	*ref*	*nc*	*nc*	*ref*
	**AL-AQ**	0% (0/18)	0·001	*nc*	0% (0-0)	0·0033	*nc*	*nc*	*nc*	*nc*
	**AL-AQ+PQ**	0% (0/19)	0·001	*nc*	0% (0-0)	0·0033	*nc*	*nc*	*nc*	*nc*
	**AS-AQ**	15·8% (3/19)	0·0002	*ref*	0% (0-0)	0·0004	*ref*	1 (1-11·9)	0·1088	*ref*
	**AS-AQ+PQ**	0% (0/20)	0·001	0·106	0% (0-0)	0·0033	0·146	*nc*	*nc*	*nc*
**Day 21**	**AL**	0% (0/20)	0·001	*ref*	0% (0-0)	·	*ref*	·	·	*ref*
	**AL-AQ**	0% (0/18)	0·001	*nc*	0% (0-0)	·	·	·	·	·
	**AL-AQ+PQ**	0% (0/18)	0·001	*nc*	0% (0-0)	·	·	·	·	·
	**AS-AQ**	5·3% (1/19)	0·0001	*ref*	0% (0-0)	0·0277	*ref*	5 (5-5)	*nc*	*ref*
	**AS-AQ+PQ**	0% (0/20)	0·001	*nc*	0% (0-0)	·	·	·	·	·
**Day 28**	**AL**	0% (0/20)	0·001	*ref*	0% (0-0)	·	*ref*	·	·	*ref*
	**AL-AQ**	0% (0/18)	0·001	*nc*	0% (0-0)	·	·	·	·	·
	**AL-AQ+PQ**	0% (0/19)	0·001	*nc*	0% (0-0)	·	·	·	·	·
	**AS-AQ**	5·3% (1/19)	0·0001	*ref*	0% (0-10·3)	0·1088	*ref*	1 (1-1)	*nc*	*ref*
	**AS-AQ+PQ**	0% (0/20)	0·001	*nc*	0% (0-0)	·	·	·	·	·

*Percentage of infectious individuals. Individuals were classed as infectious if direct membrane feeding assays (DMFA) resulted in at least one mosquito with any number of oocysts. Mosquito infection measures (mosquito infection rate and oocyst density) are presented for all participants who were infectious at baseline, and oocyst densities are from all infected mosquitoes **Mosquito infection rate is the median percentage of mosquitoes infected by each participant, where for each participant mosquito infection rate the number of mosquitoes infected as a percentage of all mosquitoes surviving to dissection. Mosquito infection rate was compared within-groups (relative to baseline) by Wilcoxon sign rank test (z-score) and between-groups by linear regression adjusted for baseline mosquito infection rate (t score, coefficient with 95% CI). ***The average oocyst density for each participant was calculated as the mean number of oocysts in infected mosquitoes (i.e., with at least one oocyst). The value presented in the table is the median of all individuals' average oocyst intensities (a composite figure of all oocysts/all infected mosquitoes is not statistically valid). P-value¥ = Within group comparison. P-value† = Between group comparison (artemether–lumefantrine vs artemether–lumefantrine-amodiaquine and artemether–lumefantrine-amodiaquine plus primaquine, artesunate-amodiaquine vs artesunate-amodiaquine plus primaquine). nc = not calculable, no positive observations. · = not tested, ref = reference group. AL = artemether-lumefantrine; AL-AQ = artemether-lumefantrine-amodiaquine; AL-AQ+PQ = artemether-lumefantrine-amodiaquine plus primaquine; AS-AQ = artesunate-amodiaquine; AS-AQ+PQ = artesunate-amodiaquine plus primaquine

Supplementary Table 3. Infectivity to mosquitoes for all individuals

Day of follow-up	Treatment arm	Mosquito infection rate Median % (IQR) *	p-value¥	p-value†	Oocyst density Median (IQR) **	p-value¥	p-value†	Median % reduction in mosquito infection rate (IQR) ***	p-value¥	p-value†
Day 0	**Overall**	2·0% (0-9·4)	·	·	1·3 (1-3·0)	·	·	·	·	·
	**AL**	3·0% (0-13·6)	*ref*	*ref*	1·7 (1-4·8)	*ref*	*ref*	·	*ref*	·
	**AL-AQ**	2·5% (0-11·2)	*ref*	0·686	1·3 (1-3·5)	*ref*	0·7563	·	*ref*	·
	**AL-AQ+PQ**	1·6% (0-4·8)	*ref*	0·071	1·2 (1-2·5)	*ref*	0·5349	·	*ref*	·
	**AS-AQ**	3·9% (1·6-19·8)	*ref*	*ref*	1·3 (1-2·3)	*ref*	*ref*	·	*ref*	·
	**AS-AQ+PQ**	1·5% (0-9·4)	*ref*	0·955	1·7 (1-3·9)	*ref*	0·7158	·	*ref*	·
Day 2	**AL**	0% (0-0)	0·0012	*ref*	1·0 (1-1)	0·1797	*ref*	88·19 (0-100)	0·001	*ref*
	**AL-AQ**	0% (0-0)	0·0012	0·513	1·0 (1-1)	0·1797	*nc*	95·30 (0-100)	0·001	0·8644
	**AL-AQ+PQ**	0% (0-0)	0·0012	0·750	*nc*	*nc*	*nc*	100 (0-100)	0·0009	0·6003
	**AS-AQ**	2·3% (0·7-8·2)	0·614	*ref*	1·2 (1-1·8)	0·8744	*ref*	7·40 (-9·40-72·91)	0·1901	*ref*
	**AS-AQ+PQ**	0% (0-0)	0·0012	0·006	*nc*	*nc*	*nc*	100 (0-100)	0·0009	0·0465
Day 7	**AL**	0% (0-0)	0·0012	*ref*	*nc*	*nc*	*ref*	100 (0-100)	0·0009	*ref*
	**AL-AQ**	0% (0-0)	0·0013	*nc*	*nc*	*nc*	*nc*	100 (0-100)	0·0009	0·707
	**AL-AQ+PQ**	0% (0-0)	0·0012	*nc*	*nc*	*nc*	*nc*	100 (0-100)	0·0009	0·8573
	**AS-AQ**	0% (0-4)	0·0011	*ref*	2·0 (1·0-2·2)	0·3991	*ref*	100 (32·38-100)	0·0018	*ref*
	**AS-AQ+PQ**	0% (0-0)	0·0012	0·070	*nc*	*nc*	*nc*	100 (0-100)	0·0009	0·5878
Day 14	**AL**	0% (0-0)	0·0012	*ref*	*nc*	*nc*	*ref*	100 (0-100)	0·0009	*ref*
	**AL-AQ**	0% (0-0)	0·0013	*nc*	*nc*	*nc*	*nc*	100 (0-100)	0·0009	0·707
	**AL-AQ+PQ**	0% (0-0)	0·0012	*nc*	*nc*	*nc*	*nc*	100 (0-100)	0·0009	0·8573
	**AS-AQ**	0% (0-0)	0·0002	*ref*	1 (1-11·9)	0·1088	*ref*	100 (90·23-100)	0·0001	*ref*
	**AS-AQ+PQ**	0% (0-0)	0·0012	0·236	*nc*	*nc*	*nc*	100 (0-100)	0·0009	0·2022
Day 21	**AL**	0% (0-0)	·	*ref*	·	·	*ref*	·	·	*ref*
	**AL-AQ**	0% (0-0)	·	·	·	·	·	·	·	·
	**AL-AQ+PQ**	0% (0-0)	·	·	·	·	·	·	·	·
	**AS-AQ**	0% (0-0)	0·022	*ref*	5 (5-5)	*nc*	*ref*	100 (77·24-100)	0·0174	*ref*
	**AS-AQ+PQ**	0% (0-0)	·	·	·	·	·	·	·	·
Day 28	**AL**	0% (0-0)	·	*ref*	·	·	*ref*	·	·	*ref*
	**AL-AQ**	0% (0-0)	·	·	·	·	·	·	·	·
	**AL-AQ+PQ**	0% (0-0)	·	·	·	·	·	·	·	·
	**AS-AQ**	0% (0-10·3)	0·1088	*ref*	1 (1-1)	*nc*	*ref*	100 (83·85-100)	0·1025	*ref*
	**AS-AQ+PQ**	0% (0-0)	·	·	·	·	·	·	·	·

Mosquito infection measure (mosquito infection rate, oocyst density and reduction in mosquito infection rate) are presented for all individuals regardless of baseline infectivity. *Mosquito infection rate is the median percentage of mosquitoes infected by each participant, where for each participant mosquito infection rate the number of mosquitoes infected as a percentage of all mosquitoes surviving to dissection. Mosquito infection rate was compared within-groups (relative to baseline) by Wilcoxon sign rank test (z-score) and between-groups by linear regression adjusted for baseline mosquito infection rate (t score, coefficient with 95% CI). **The average oocyst density for each participant was calculated as the mean number of oocysts in infected mosquitoes (i.e., with at least one oocyst). The value presented in the table is the median of all individuals' average oocyst intensities (a composite figure of all oocysts/all infected mosquitoes is not statistically valid). *** Median within-person (relative to baseline) reduction in mosquito infection, including individuals not infectious at baseline. P-value¥ = Within group comparison. P-value† = Between group comparison (artemether–lumefantrine vs artemether–lumefantrine-amodiaquine and artemether–lumefantrine-amodiaquine plus primaquine, artesunate-amodiaquine vs artesunate-amodiaquine plus primaquine). nc = not calculable, no positive observations. · = not tested, ref = reference group. AL = artemether-lumefantrine; AL-AQ = artemether-lumefantrine-amodiaquine; AL-AQ+PQ = artemether-lumefantrine-amodiaquine plus primaquine; AS-AQ = artesunate-amodiaquine; AS-AQ+PQ = artesunate-amodiaquine plus primaquine

Supplementary Figure 2. Difference in mosquito infection rate and person infectivity before and after gametocyte enrichment

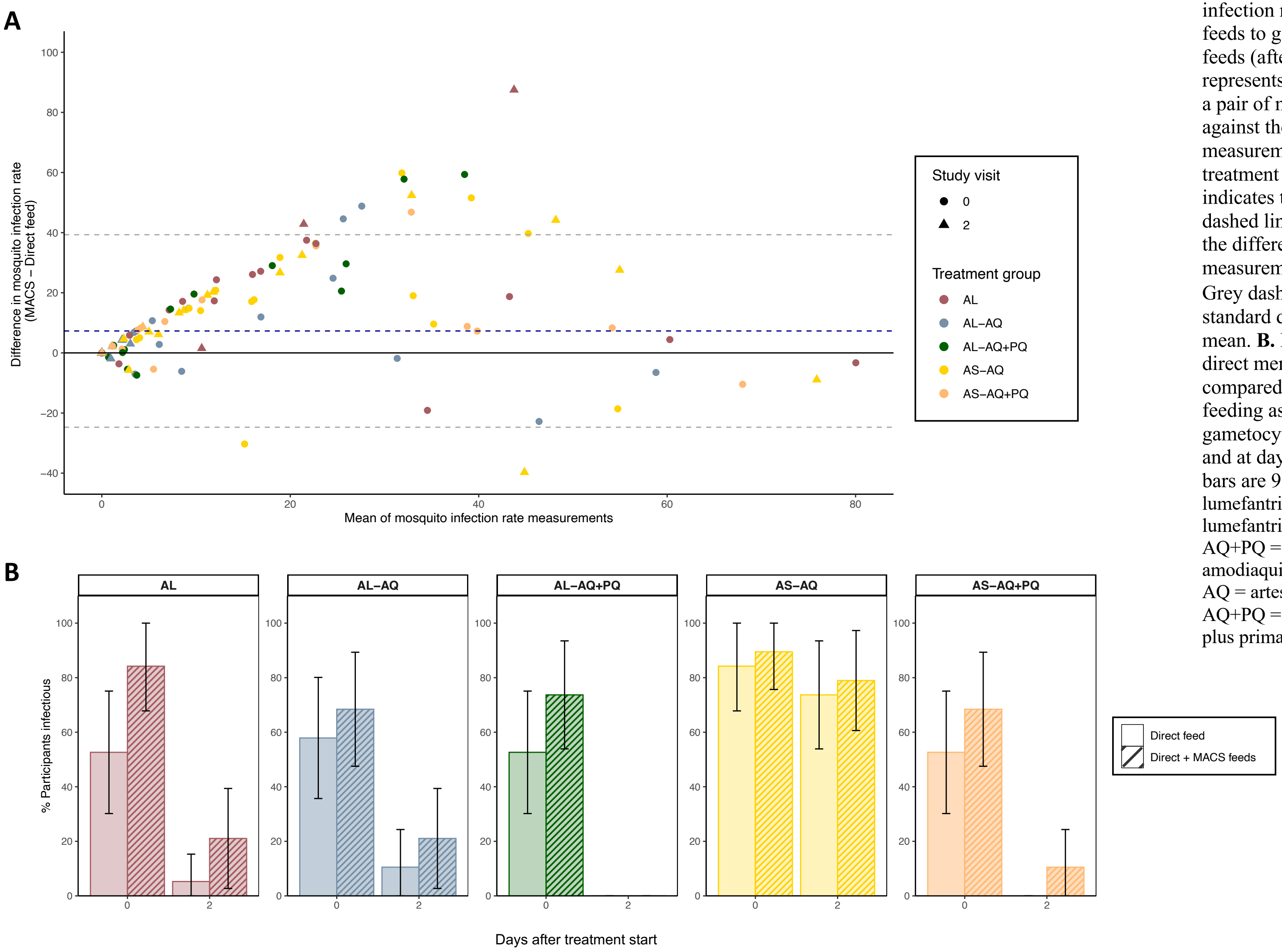

A. Difference in mosquito infection rate comparing direct feeds to gametocyte enriched feeds (after MACS). Each point represents the difference between a pair of measurements plotted against the average of the two measurements. Colour indicates treatment group and shape indicates the study visit. Blue dashed line indicates the mean of the difference between measurements across all samples. Grey dashed lines represent ± 1·96 standard deviations (SD) of the mean. **B.** Person infectivity by direct membrane feeding assay compared to direct membrane feeding assay boosted by gametocyte enrichment at baseline and at day 2 post-treatment. Error bars are 95% CI. AL = artemether-lumefantrine; AL-AQ = artemether-lumefantrine-amodiaquine; AL-AQ+PQ = artemether-lumefantrine-amodiaquine plus primaquine; AS-AQ = artesunate-amodiaquine; AS-AQ+PQ = artesunate-amodiaquine plus primaquine

Supplementary Table 4. Participant infectivity after gametocyte enrichment.

	Direct membrane feed	Direct and gametocyte enriched membrane feeds		
	**Infectious individuals % (n/N)**	**Infectious individuals % (n/N)**	**p-value[¥]**	**p-value†**
**Day 0**				
**AL**	53% (10/19)	84% (16/19)	0·039	*ref*
**AL-AQ**	58% (11/19)	68% (13/19)	0·369	0·224
**AL-AQ+PQ**	53% (10/19)	74% (14/19)	0·157	0·695
**AS-AQ**	84% (16/19)	89% (17/19)	0·500	*ref*
**AS-AQ+PQ**	53% (10/19)	68% (13/19)	0·254	0·232
**Day 2**				
**AL**	5% (1/19)	21% (4/19)	0·170	*ref*
**AL-AQ**	11% (2/19)	21% (4/19)	0·330	0·654
**AL-AQ+PQ**	0% (0/18)	0% (0/18)	nc	0·059
**AS-AQ**	74% (14/19)	79% (15/19)	0·500	*ref*
**AS-AQ+PQ**	0% (0/19)	11% (2/19)	0·243	<0·0001

Participant infectivity after direct membrane feeding assay and gametocyte enrichment-boosted direct membrane feeding assays at baseline and day 2 after treatment initiation. [¥]Within-group comparison (direct feed as reference). [†]Between artemisinin-based combination therapy matched group comparison (i.e., artemether–lumefantrine vs artemether–lumefantrine-amodiaquine and artemether–lumefantrine-amodiaquine plus primaquine, artesunate-amodiaquine vs artesunate-amodiaquine plus primaquine) by Fishers exact test. Nc = not calculable, ref = reference group. AL = artemether-lumefantrine; AL-AQ = artemether-lumefantrine-amodiaquine; AL-AQ+PQ = artemether-lumefantrine-amodiaquine plus primaquine; AS-AQ = artesunate-amodiaquine; AS-AQ+PQ = artesunate-amodiaquine plus primaquine

Supplementary Table 5. Asexual parasite density by microscopy

Day of follow-up	Treatment arm	Median asexual parasites/µL (IQR)	p-value¥	p-value†	Prevalence n/N (%)	p-value¥	p-value†
Day 0	**Overall**	0·00 (0·00-241·99)	·	·	37% (37/96)	·	·
	**AL**	37·9 (0·0-300·0)	*ref*	*ref*	50% (10/20)	*ref*	*ref*
	**AL-AQ**	0·0 (0·0-79·8)	*ref*	0·1193	30% (6/20)	*ref*	0·167
	**AL-AQ+PQ**	0·0 (0·0-37·6)	*ref*	0·0871	25% (5/20)	*ref*	0·095
	**AS-AQ**	0·0 (0·0-1654·9)	*ref*	*ref*	40% (8/20)	*ref*	*ref*
	**AS-AQ+PQ**	0·0 (0·0-720·0)	*ref*	0·5824	40% (8/20)	*ref*	0·626
Day 2	**AL**	0·0 (0·0-0·0)	0·0031	*ref*	5% (1/20)	0·004	*ref*
	**AL-AQ**	0·0 (0·0-0·0)	0·0148	0·3297	0% (0/19)	0·031	0·513
	**AL-AQ+PQ**	0·0 (0·0-0·0)	0·0459	0·3297	0% (0/19)	0·125	0·513
	**AS-AQ**	0·0 (0·0-0·0)	0·0051	*ref*	5% (1/20)	0·016	*ref*
	**AS-AQ+PQ**	0·0 (0·0-0·0)	0·0051	0·3173	0% (0/20)	0·008	0·500
Day 7	**AL**	0·0 (0·0-0·0)	0·0019	*ref*	0% (0/20)	0·002	*ref*
	**AL-AQ**	0·0 (0·0-0·0)	0·0149	nc	0% (0/18)	0·031	nc
	**AL-AQ+PQ**	0·0 (0·0-0·0)	0·0459	nc	0% (0/19)	0·125	nc
	**AS-AQ**	0·0 (0·0-0·0)	0·0051	*ref*	0% (0/20)	0·008	*ref*
	**AS-AQ+PQ**	0·0 (0·0-0·0)	0·0051	nc	0% (0/20)	0·008	nc
Day 14	**AL**	0·0 (0·0-0·0)	0·0019	*ref*	0% (0/20)	0·002	*ref*
	**AL-AQ**	0·0 (0·0-0·0)	0·0149	nc	0% (0/18)	0·031	nc
	**AL-AQ+PQ**	0·0 (0·0-0·0)	0·0459	nc	0% (0/19)	0·125	nc
	**AS-AQ**	0·0 (0·0-0·0)	0·0087	*ref*	0% (0/19)	0·008	*ref*
	**AS-AQ+PQ**	0·0 (0·0-0·0)	0·0051	nc	0% (0/20)	0·008	nc
Day 21	**AL**	0·0 (0·0-0·0)	0·0019	*ref*	0% (0/20)	0·002	*ref*
	**AL-AQ**	0·0 (0·0-0·0)	0·0149	nc	0% (0/18)	0·031	nc
	**AL-AQ+PQ**	0·0 (0·0-0·0)	0·0836	nc	0% (0/18)	0·125	nc
	**AS-AQ**	0·0 (0·0-0·0)	0·0087	*ref*	0% (0/19)	0·008	*ref*
	**AS-AQ+PQ**	0·0 (0·0-0·0)	0·0051	nc	0% (0/20)	0·008	nc
Day 28	**AL**	0·0 (0·0-0·0)	0·0139	*ref*	5% (1/20)	0·002	*ref*
	**AL-AQ**	0·0 (0·0-0·0)	0·0149	0·3428	0% (0/18)	0·031	0·526
	**AL-AQ+PQ**	0·0 (0·0-0·0)	0·0459	0·3297	0% (0/19)	0·125	0·513
	**AS-AQ**	0·0 (0·0-0·0)	0·0087	*ref*	0% (0/19)	0·008	*ref*
	**AS-AQ+PQ**	0·0 (0·0-0·0)	0·0051	nc	0% (0/20)	0·008	nc

Asexual parasite density (asexual parasites / µL) and prevalence of asexual parasites at all time points, measured by thick film microscopy (counted against 200 WBC). ¥Within-group comparisons †Between artemisinin-based combination therapy matched group comparison (i.e., artemether–lumefantrine vs artemether–lumefantrine-amodiaquine and artemether–lumefantrine-amodiaquine plus primaquine, artesunate-amodiaquine vs artesunate-amodiaquine plus primaquine). Nc = not calculable, ref = reference group. AL = artemether-lumefantrine; AL-AQ = artemether-lumefantrine-amodiaquine; AL-AQ+PQ = artemether-lumefantrine-amodiaquine plus primaquine; AS-AQ = artesunate-amodiaquine; AS-AQ+PQ = artesunate-amodiaquine plus primaquine

Supplementary Figure 3. Gametocyte density and prevalence by gametocyte sex

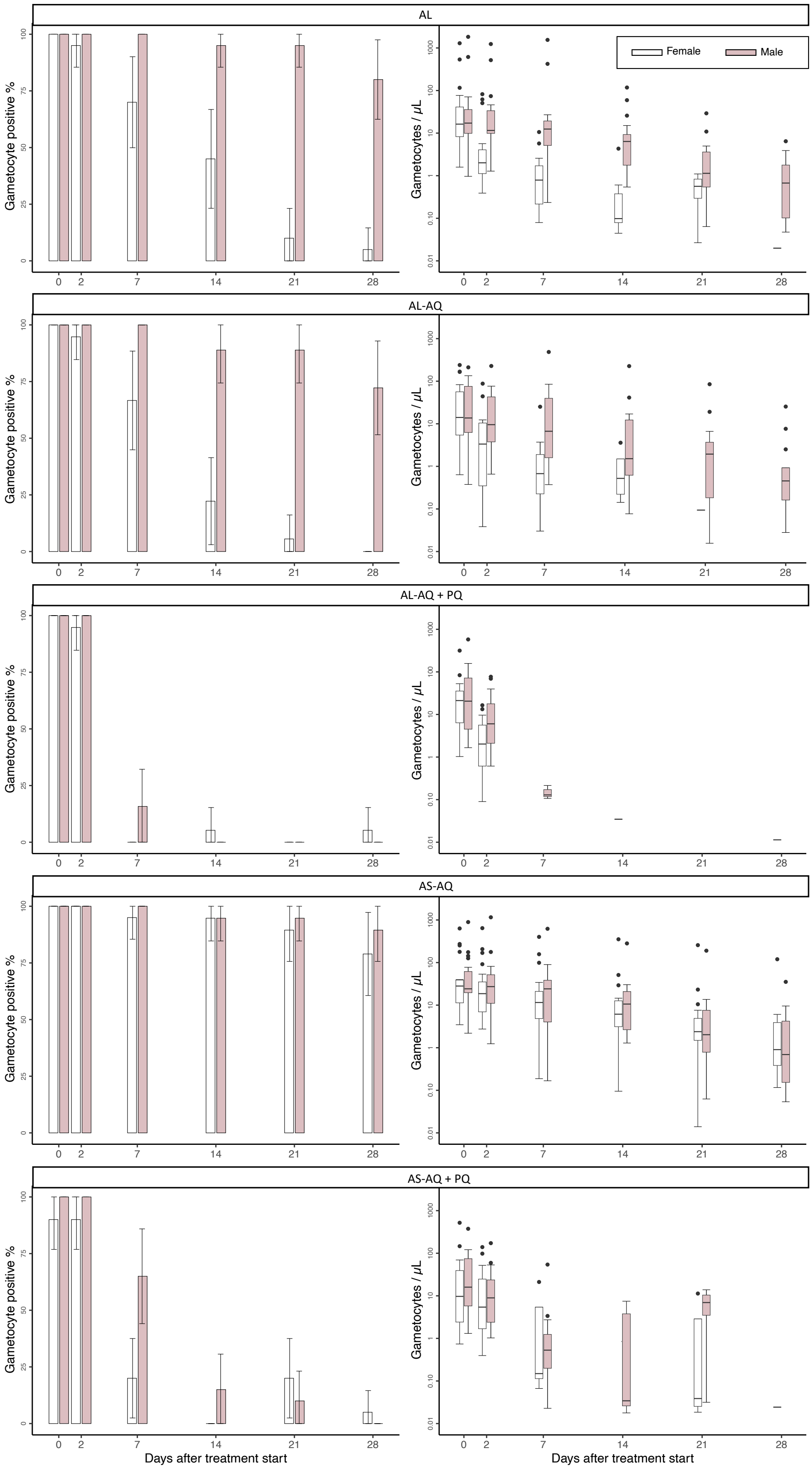

AL = artemether-lumefantrine; AL-AQ = artemether-lumefantrine-amodiaquine; AL-AQ+PQ = artemether-lumefantrine-amodiaquine plus primaquine; AS-AQ = artesunate-amodiaquine; AS-AQ+PQ = artesunate-amodiaquine plus primaquine

Supplementary Table 6. Total gametocyte density, prevalence and sex ratio

Day of follow-up	Total gametocytes (CCP4 & PfMGET)						
	Treatment arm	Median gametocytes/µL (IQR)	p-value	Prevalence n/N (%)	p-value	Proportion male Median (IQR)	p-value
Day 0	**Overall**	38·01 (13·55-113·01)	·	100% (100/100)	·	0·54 (0·42-0·65)	·
	**AL**	30·96 (19·90-92·72)	*ref*	100% (20/20)	*ref*	0·50 (0·42-0·65)	*ref*
	**AL-AQ**	28·58 (11·49-130·45)	0·908	100% (20/20)	*nc*	0·53 (0·42-0·62)	0·9784
	**AL-AQ+PQ**	42·30 (11·79-97·04)	0·838	100% (20/20)	*nc*	0·52 (0·43-0·70)	0·3302
	**AS-AQ**	52·52 (33·59-128·97)	*ref*	100% (20/20)	*ref*	0·52 (0·37-0·64)	*ref*
	**AS-AQ+PQ**	24·82 (10·74-115·22)	0·105	100% (20/20)	*nc*	0·63 (0·49-0·68)	0·1231
Day 2	**AL**	15·43 (10·30-43·69)	*ref*	100% (20/20)	*ref*	0·88 (0·77-0·94)	*ref*
	**AL-AQ**	11·39 (4·12-59·23)	0·684	100% (19/19)	*nc*	0·82 (0·77-0·92)	0·4397
	**AL-AQ+PQ**	7·77 (3·43-25·28)	0·066	100% (19/19)	*nc*	0·77 (0·68-0·93)	0·1559
	**AS-AQ**	45·56 (19·18-100·32)	*ref*	100% (20/20)	*ref*	0·50 (0·44-0·67)	*ref*
	**AS-AQ+PQ**	12·64 (5·11-49·64)	0·011	100% (20/20)	*nc*	0·54 (0·41-0·71)	0·7251
Day 7	**AL**	13·25 (5·61-21)	*ref*	100% (20/20)	*ref*	0·98 (0·91-1)	*ref*
	**AL-AQ**	6·89 (1·05-49·86)	0·937	100% (18/18)	*nc*	0·99 (0·93-1)	0·6775
	**AL-AQ+PQ**	0 (0-0)	0·002	15·8% (3/19)	<0·0001	1 (1-1)	0·2389
	**AS-AQ**	31·74 (7·27-61·59)	*ref*	100% (20/20)	*ref*	0·59 (0·36-0·73)	*ref*
	**AS-AQ+PQ**	0·17 (0-0·87)	<0·0001	70·0% (14/20)	0·010	1 (0·88-1)	0·0002
Day 14	**AL**	5·61 (1·41-9·30)	*ref*	95·0% (19/20)	*ref*	1 (0·98-1)	*ref*
	**AL-AQ**	1·44 (0·37-11·63)	0·899	88·9% (16/18)	0·459	1 (1-1)	0·2648
	**AL-AQ+PQ**	0 (0-0)	0·246	5·3% (1/19)	<0·0001	*nc*	*nc*
	**AS-AQ**	13·65 (4·93-32)	*ref*	94·7% (18/19)	*ref*	0·49 (0·38-0·68)	*ref*
	**AS-AQ+PQ**	0 (0-0)	0·014	15·0% (3/20)	<0·0001	1 (1-1)	0·1003
Day 21	**AL**	1·02 (0·50-3·60)	*ref*	95·0% (19/20)	*ref*	1 (1-1)	*ref*
	**AL-AQ**	1·16 (0·09-3·44)	0·212	88·9% (16/18)	0·459	1 (1-1)	0·7063
	**AL-AQ+PQ**	0 (0-0)	*nc*	0% (0/18)	<0·0001	*nc*	*nc*
	**AS-AQ**	4·25 (0·91-13·39)	*ref*	94·7% (18/19)	*ref*	0·47 (0·37-0·72)	*ref*
	**AS-AQ+PQ**	0 (0-0)	0·500	20·0% (4/20)	<0·0001	0·55 (0·55-0·55)	0·6299
Day 28	**AL**	0·21 (0·06-1·48)	*ref*	80·0% (16/20)	*ref*	1 (1-1)	*ref*
	**AL-AQ**	0·21 (0-0·63)	0·307	72·2% (13/18)	0·427	1 (1-1)	*nc*
	**AL-AQ+PQ**	0 (0-0)	0·579	5·3% (1/19)	<0·0001	*nc*	*nc*
	**AS-AQ**	1·22 (0·22-7·23)	*ref*	94·7% (18/19)	*ref*	0·42 (0·27-0·90)	*ref*
	**AS-AQ+PQ**	0 (0-0)	0·543	5·0% (1/20)	<0·0001	*nc*	*nc*

P-values are for differences between artemisinin-based combination therapy matched group comparison (i.e., artemether–lumefantrine vs artemether–lumefantrine-amodiaquine and artemether–lumefantrine-amodiaquine plus primaquine, artesunate-amodiaquine vs artesunate-amodiaquine plus primaquine). Density was compared using regression analyses of log10 transformed density values, with adjustment for baseline densities. Prevalence was compared with one sided Fishers exact tests. For males and females, proportion male is given for participants/time-points with total gametocyte densities of 0·2/µL and over, as described previously (1). For the calculation of gametocyte prevalence, samples were classified as negative for a particular gametocyte sex if the estimated density of in gametocytes of that sex was less than 0·01/µL (i.e. one gametocyte per 100 µL of blood sample). P-value = between group comparison. nc = not calculable, no observations/no observations over the threshold density for analysis, ·= not tested, *ref* = reference group, AL = artemether-lumefantrine; AL-AQ = artemether-lumefantrine-amodiaquine; AL-AQ+PQ = artemether-lumefantrine-amodiaquine plus primaquine; AS-AQ = artesunate-amodiaquine; AS-AQ+PQ = artesunate-amodiaquine plus primaquine.

Supplementary Table 7. Gametocyte circulation time and area under the curve

	Treatment group	Total gametocytes (CCP4 & PfMGET)	p-value*	Female gametocytes (CCP4)	p-value*	Male gametocytes (PfMGET)	p-value*	p-value♂♀
Circulation time Days (95% CI)	AL	6·13 (5·36-6·90)	*ref*	3·19 (2·69-3·70)	*ref*	6·83 (5·95-7·72)	*ref*	<0·0001
	AL-AQ	6·00 (5·20-6·79)	0·8071	2·75 (2·17-3·32)	0·2410	6·75 (5·82-7·68)	0·8951	<0·0001
	AL-AQ+PQ	2·60 (2·06-3·13)	<0·0001	3·27 (2·31-4·24)	0·8824	1·31 (1·00-1·63)	<0·0001	0·0006
	AS-AQ	7·99 (6·70-9·28)	*ref*	9·07 (7·16-10·98)	*ref*	7·77 (6·63-8·91)	*ref*	0·0066
	AS-AQ+PQ	3·30 (2·79-3·81)	<0·0001	4·81 (3·45-6·17)	0·0005	3·43 (2·76-4·11)	<0·0001	0·1191
AUC Median (IQR) gametocytes per uL/day	AL	9·36 (5·31-21·91)	*ref*	0·88 (0·61-2·21)	*ref*	7·79 (3·96-17·55)	*ref*	<0·0001
	AL-AQ	5·35 (2·21-40·44)	0·323	1·11 (0·33-3·02)	0·925	3·55 (1·54-26·71)	0·071	<0·0001
	AL-AQ+PQ	4·42 (1·22-14·86)	<0·0001	1·15 (0·31-2·69)	0·057	2·42 (0·75-10·78)	<0·0001	<0·0001
	AS-AQ	26·39 (12·80-51·70)	*ref*	7·23 (5·03-23·10)	*ref*	19·64 (4·83-27·47)	*ref*	0·0337
	AS-AQ+PQ	6·03 (2·84-18·41)	0·002	1·35 (0·23-5·84)	0·001	3·19 (2·27-11·11)	0·007	0·0003

Gametocyte circulation time was calculated using a deterministic compartmental model (5), and is presented as the model estimate (mean days) with 95% CI. Area under the curve (AUC) of gametocyte density per participant over time was calculated using the linear trapezoid method (6), and is presented as the median and IQR of individual AUC values by treatment arm. P-values are for differences in the t-statistic between AL-AQ, AL-AQ+PQ and the AL reference group, and between AS-AQ+PQ and the AS-AQ reference group (*), and for between sexes within treatment groups (♂♀). *Ref* = reference, AL = artemether-lumefantrine; AL-AQ = artemether-lumefantrine-amodiaquine; AL-AQ+PQ = artemether-lumefantrine-amodiaquine plus primaquine; AS-AQ = artesunate-amodiaquine; AS-AQ+PQ = artesunate-amodiaquine plus primaquine.

Supplementary Table 8. Female (CCP4) and male (PfMGET) gametocyte density and prevalence

Day of follow-up	Treatment arm	Female gametocytes (CCP4)				Male gametocytes (PfMGET)			
		Median/µL (IQR)	p-value	Prevalence n/N (%)	p-value	Median/µL (IQR)	p-value	Prevalence n/N (%)	p-value
Day 0	Overall	15·63 (6·12-39·20)	·	98% (98/100)	·	19·72 (8·52-69·39)	·	100% (100/100)	·
	AL	16·26 (7·83-47·60)	*ref*	100% (20/20)	*ref*	17·19 (9·52-39·56)	*ref*	100% (20/20)	*ref*
	AL-AQ	14·07 (4·87-61·79)	0·890	100% (20/20)	*nc*	13·70 (6·20-77·32)	0·825	100% (20/20)	*nc*
	AL-AQ+PQ	21·19 (5·73-35·53)	0·935	100% (20/20)	*nc*	20·54 (4·44-75·63)	0·637	100% (20/20)	*nc*
	AS-AQ	28·10 (11·26-39·20)	*ref*	100% (20/20)	*ref*	24·18 (19·48-66·89)	*ref*	100% (20/20)	*ref*
	AS-AQ+PQ	7·47 (1·43-38·49)	0·090	90% (18/20)	0·244	15·98 (5·08-76·78)	0·234	100% (20/20)	*nc*
Day 2	AL	1·81 (0·98-4·05)	*ref*	95% (19/20)	*ref*	11·56 (9·69-35·11)	*ref*	100% (20/20)	*ref*
	AL-AQ	2·35 (0·29-10·90)	0·207	94·7% (18/19)	0·744	9·55 (3·60-48·33)	0·701	100% (19/19)	*nc*
	AL-AQ+PQ	1·95 (0·46-6·23)	0·880	94·7% (18/19)	0·744	6·03 (1·94-18·23)	0·040	100% (19/19)	*nc*
	AS-AQ	18·61 (6·51-41·24)	*ref*	100% (20/20)	*ref*	27·25 (10·53-57·52)	*ref*	100% (20/20)	*ref*
	AS-AQ+PQ	4·35 (1·36-22·11)	0·018	90% (18/20)	0·244	8·94 (2·29-28·44)	0·024	100% (20/20)	*nc*
Day 7	AL	0·26 (0-1·23)	*ref*	70% (14/20)	*ref*	12·46 (4·87-20·28)	*ref*	100% (20/20)	*ref*
	AL-AQ	0·18 (0-1·17)	0·566	66·7% (12/18)	0·550	6·68 (1·05-46·15)	1·000	100% (18/18)	*nc*
	AL-AQ+PQ	0 (0-0)	*nc*	0% (0/19)	<0·0001	0 (0-0)	0·003	15·8% (3/19)	<0·0001
	AS-AQ	11·07 (4·06-21·03)	*ref*	95% (19/20)	*ref*	24·15 (3·78-39·47)	*ref*	100% (20/20)	*ref*
	AS-AQ+PQ	0 (0-0)	0·064	20% (4/20)	<0·0001	0·17 (0-0·87)	0·00042	65% (13/20)	0·004
Day 14	AL	0 (0-0·10)	*ref*	45% (9/20)	*ref*	5·61 (1·41-9·26)	*ref*	95% (19/20)	*ref*
	AL-AQ	0 (0-0)	0·668	22·2% (4/18)	0·128	1·44 (0·37-11·38)	0·908	88·9% (16/18)	0·459
	AL-AQ+PQ	0 (0-0)	0·729	5·3% (1/19)	0·005	0 (0-0)	*nc*	0% (0/19)	<0·0001
	AS-AQ	5·61 (2·99-12·68)	*ref*	94·7% (18/19)	*ref*	10·23 (1·68-21·99)	*ref*	94·7% (18/19)	*ref*
	AS-AQ+PQ	0 (0-0)	*nc*	0% (0/20)	<0·0001	0 (0-0)	0·115	15% (3/20)	<0·0001
Day 21	AL	0 (0-0)	*ref*	10·0% (2/20)	*ref*	1·02 (0·50-3·6)	*ref*	95% (19/20)	*ref*
	AL-AQ	0 (0-0)	*nc*	5·6% (1/18)	0·541	1·16 (0·09-3·44)	0·199	88·9% (16/18)	0·459
	AL-AQ+PQ	0 (0-0)	*nc*	0% (0/18)	0·270	0 (0-0)	*nc*	0% (0/18)	<0·0001
	AS-AQ	2·24 (0·28-4·89)	*ref*	89·5% (17/19)	*ref*	2·01 (0·72-7·79)	*ref*	94·7% (18/19)	*ref*
	AS-AQ+PQ	0 (0-0)	0·832	20·0% (4/20)	<0·0001	0 (0-0)	0·512	10% (2/20)	<0·0001
Day 28	AL	0 (0-0)	*ref*	5·0% (1/20)	*ref*	0·21 (0·05-1·48)	*ref*	80% (16/20)	*ref*
	AL-AQ	0 (0-0)	*nc*	0% (0/18)	0·526	0·21 (0-0·63)	0·310	72·2% (13/18)	0·427
	AL-AQ+PQ	0 (0-0)	*nc*	5·3% (1/19)	0·744	0 (0-0)	*nc*	0% (0/19)	<0·0001
	AS-AQ	0·45 (0·12-3·21)	*ref*	78·9% (15/19)	*ref*	0·44 (0·13-4·21)	*ref*	89·5% (17/19)	*ref*
	AS-AQ+PQ	0 (0-0)	0·663	5·0% (1/20)	<0·0001	0 (0-0)	*nc*	0% (0/20)	<0·0001

P-values are for differences between artemisinin-based combination therapy matched group comparison (i.e., artemether–lumefantrine vs artemether–lumefantrine-amodiaquine and artemether–lumefantrine-amodiaquine plus primaquine, artesunate-amodiaquine vs artesunate-amodiaquine plus primaquine). Density was compared using regression analyses of log10 transformed density values, with adjustment for baseline densities. Prevalence was compared with one sided Fishers exact tests. For the calculation of gametocyte prevalence, samples were classified as negative for a particular gametocyte sex if the estimated density of in gametocytes of that sex was less than 0·01 gametocytes per µL (i.e. one gametocyte per 100 µL of blood sample). · = not tested. *ref* = reference group, *nc* = not calculable. AL = artemether-lumefantrine; AL-AQ = artemether-lumefantrine-amodiaquine; AL-AQ+PQ = artemether-lumefantrine-amodiaquine plus primaquine; AS-AQ = artesunate-amodiaquine; AS-AQ+PQ = artesunate-amodiaquine plus primaquine

Supplementary Figure 4. Proportion of gametocytes that were male

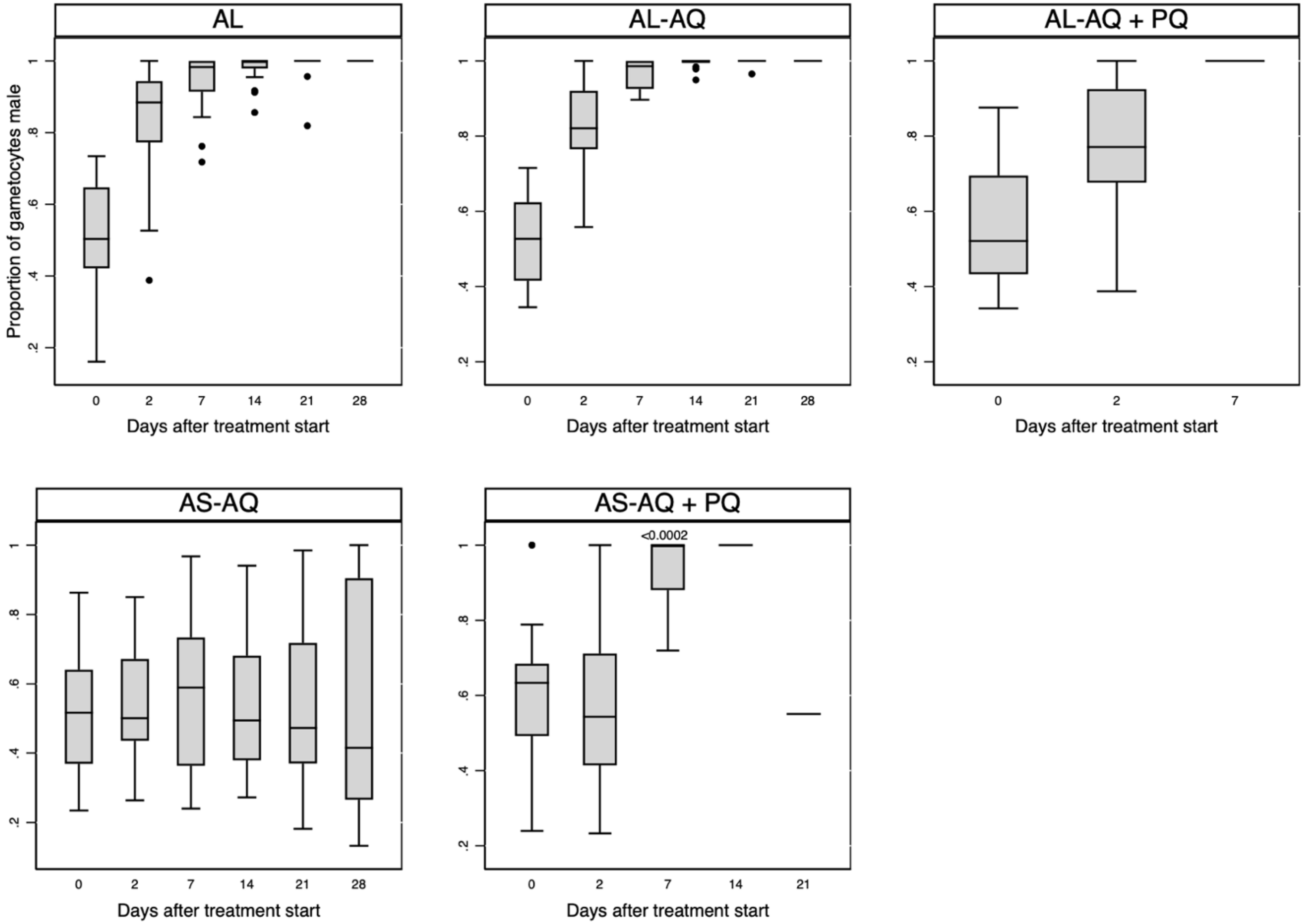

The proportion of gametocytes that were male was calculated for all values with total gametocyte densities of 0.2/µL and over, as described previously.(1) P-values (<0.05) for differences between treatment groups AL-AQ, AL-AQ+PQ and the AL reference group, and between AS-AQ+PQ and the AS-AQ reference group, were calculated using Wilcoxon rank sum tests. AL = artemether-lumefantrine; AL-AQ = artemether-lumefantrine-amodiaquine; AL-AQ+PQ = artemether-lumefantrine-amodiaquine plus primaquine; AS-AQ = artesunate-amodiaquine; AS-AQ+PQ = artesunate-amodiaquine plus primaquine.

Supplementary Table 9. Gametocyte infectivity

Day of follow-up	Treatment arm	Odds ratio (95% CI)	P-value
Day 0	AL	1	*ref*
	AL-AQ	0·64 (0·47-0·89)	0·008
	AL-AQ+PQ	0·19 (0·13-0·28)	<0·0001
	AS-AQ	1	*ref*
	AS-AQ+PQ	1·71 (1·29-2·27)	<0·0001
Day 2	AL	1	*ref*
	AL-AQ	1·3 (0·15-11·27)	0·808
	AL-AQ+PQ	*nc*	*nc*
	AS-AQ	1	*ref*
	AS-AQ+PQ	*nc*	*nc*

Odds ratios are for the change in mosquito infection rate in the ALAQ and ALAQ+PQ groups compared to the reference (AL) group and the AS-AQ+PQ group compared to the reference (AS-AQ) group with adjustment for gametocyte densities. At later timepoints, there were too few infected mosquitoes to calculate the odd ratios. *nc* = not calculable, no observations (too few infected mosquitoes for convergence), *ref* = reference, AL = artemether-lumefantrine; AL-AQ = artemether-lumefantrine-amodiaquine; AL-AQ+PQ = artemether-lumefantrine-amodiaquine plus primaquine; AS-AQ = artesunate-amodiaquine; AS-AQ+PQ = artesunate-amodiaquine plus primaquine.

Supplementary Table 10. Haemoglobin density

Day of follow-up	Treatment arm	Mean g/dL (range)	p-value¥	p-value†	Percent change from day 0			
					Mean (lower/upper 95% CI)	Range	p-value¥	p-value†
Day 0	**Overall**	12·0 (10·1-14·9)	·	·	·	·	·	·
	**AL**	12·5 (10·4-14·9)	*ref*	*ref*	·	·	·	·
	**AL-AQ**	12 (10·1-14·9)	*ref*	0·283	·	·	·	·
	**AL-AQ+PQ**	11·7 (10·4-13·4)	*ref*	0·031	·	·	·	·
	**AS-AQ**	11·8 (10·5-14·5)	*ref*	*ref*	·	·	·	·
	**AS-AQ+PQ**	11·9 (10·1-13·8)	*ref*	0·748	·	·	·	·
Day 1	**AL**	11·9 (9·5-15·5)	0·0354	*ref*	-4·41% (-8·28 / -0·54)	-25·17/8·65	0·0278	*ref*
	**AL-AQ**	11·7 (10-15)	0·1068	0·530	-2·40% (-5·52 / 0·71)	-12·03/8·18	0·1232	0·4038
	**AL-AQ+PQ**	11·1 (9·8-14)	0·0005	0·761	-5·36% (-7·93 / -2·79)	-15·97/4·48	0·0003	0·6719
	**AS-AQ**	11·4 (9-15·5)	0·0069	*ref*	-3·79% (-6·42 / -1·17)	-14·29/7·62	0·0070	*ref*
	**AS-AQ+PQ**	11·5 (9·6-13·6)	0·0408	0·821	-3·19% (-6·35 / -0·04)	-13·79/8·13	0·0474	0·7617
Day 2	**AL**	12 (10·1-15)	0·0006	*ref*	-4·02% (-6·02 / -2·02)	-14·50/4·20	0·0005	*ref*
	**AL-AQ**	11·5 (9·6-13·2)	0·0007	0·411	-4·83% (-7·25 / -2·41)	-12·71/4·55	0·0005	0·5890
	**AL-AQ+PQ**	11 (9·6-12·5)	<0·00001	0·167	-5·42% (-7·18 / -3·66)	-12·31/3·67	<0·00001	0·2779
	**AS-AQ**	11·2 (9·9-13·5)	<0·00001	*ref*	-5·59% (-7·54 / -3·64)	-14·63/1·74	<0·00001	*ref*
	**AS-AQ+PQ**	11·4 (10·2-13·6)	0·0005	0·288	-4·36% (-6·50 / -2·23)	-13·33/4·27	0·0004	0·3797
Day 7	**AL**	12·3 (10·1-15·9)	0·1394	*ref*	-1·52% (-3·66 / 0·62)	-11·40/6·71	0·1543	*ref*
	**AL-AQ**	12 (10·4-14·2)	0·5061	0·947	-0·55% (-3·40 / 2·31)	-7·52/16·35	0·6917	0·5649
	**AL-AQ+PQ**	11·6 (10·4-13·7)	0·7899	0·629	-0·23% (-2·30 / 1·84)	-12·61/5·50	0·8165	0·3715
	**AS-AQ**	11·6 (9·7-14·2)	0·1751	*ref*	-1·53% (-4·05 / 0·99)	-13·01/8·33	0·2191	*ref*
	**AS-AQ+PQ**	11·8 (10·2-13·3)	0·1723	0·802	-1·31% (-3·61 / 0·99)	-10·53/6·25	0·2478	0·8933
Day 14	**AL**	12·6 (10·5-15·5)	0·4351	*ref*	1·37% (-1·50 / 4·23)	-10·29/13·21	0·3309	*ref*
	**AL-AQ**	12·3 (11·1-14·6)	0·2260	0·787	1·86% (-0·85 / 4·57)	-8·46/11·54	0·1657	0·7948
	**AL-AQ+PQ**	11·9 (10·9-13·2)	0·1487	0·568	2·40% (-0·58 / 5·38)	-10·77/11·30	0·1082	0·6030
	**AS-AQ**	11·9 (10·4-14·8)	0·2327	*ref*	1·19% (-0·63 / 3·00)	-4·07/7·62	0·1869	*ref*
	**AS-AQ+PQ**	12·5 (10·5-13·8)	0·0026	0·021	4·64% (1·90 / 7·37)	-6·67/19·27	0·0021	0·0357
Day 21	**AL**	12·6 (10·6-15·2)	0·5576	*ref*	1·65% (-2·79 / 6·09)	-10·92/21·70	0·4462	*ref*
	**AL-AQ**	12·6 (11-14·7)	0·0208	0·481	4·60% (0·98 / 8·21)	-4·17/25·64	0·0157	0·2938
	**AL-AQ+PQ**	12·1 (11·1-13·4)	0·0092	0·943	4·34% (1·41 / 7·27)	-9·23/12·50	0·0062	0·3075
	**AS-AQ**	12·2 (10·3-14·9)	0·0429	*ref*	3·77% (0·48 / 7·07)	-14·39/18·10	0·0272	*ref*
	**AS-AQ+PQ**	12·3 (10·3-13·6)	0·0087	0·923	3·06% (0·89 / 5·23)	-2·68/11·93	0·0083	0·7026
Day 28	**AL**	12·8 (11·3-15·2)	0·0838	*ref*	2·75% (-0·05 / 5·55)	-7·69/16·04	0·0535	*ref*
	**AL-AQ**	12·7 (10·6-14·5)	0·0103	0·436	5·33% (1·50 / 9·17)	-9·40/18·18	0·0093	0·2540
	**AL-AQ+PQ**	12·1 (11·3-13·1)	0·0233	0·266	3·71% (0·62 / 6·80)	-5·04/21·15	0·0213	0·6328
	**AS-AQ**	12·4 (11·1-14·9)	0·0009	*ref*	5·67% (2·64 / 8·71)	-1·63/19·64	0·0010	*ref*
	**AS-AQ+PQ**	12·5 (10·8-14·3)	0·0029	0·850	4·86% (1·83 / 7·88)	-4·31/15·60	0·0033	0·6914

Haemoglobin density and percent reduction in haemoglobin density (relative to baseline) were compared within treatment arms (p-value¥) using paired t-tests (with day 0 as reference for percent change) and between treatment arms (p-value†) using linear regression (for density, adjusted for baseline Hb density) or two-way t-tests (for percent reduction). · = not tested. *ref* = reference group, AL = artemether-lumefantrine; AL-AQ = artemether-lumefantrine-amodiaquine; AL-AQ+PQ = artemether-lumefantrine-amodiaquine plus primaquine; AS-AQ = artesunate-amodiaquine; AS-AQ+PQ = artesunate-amodiaquine plus primaquine.

Supplementary Figure 5. Absolute haemoglobin density

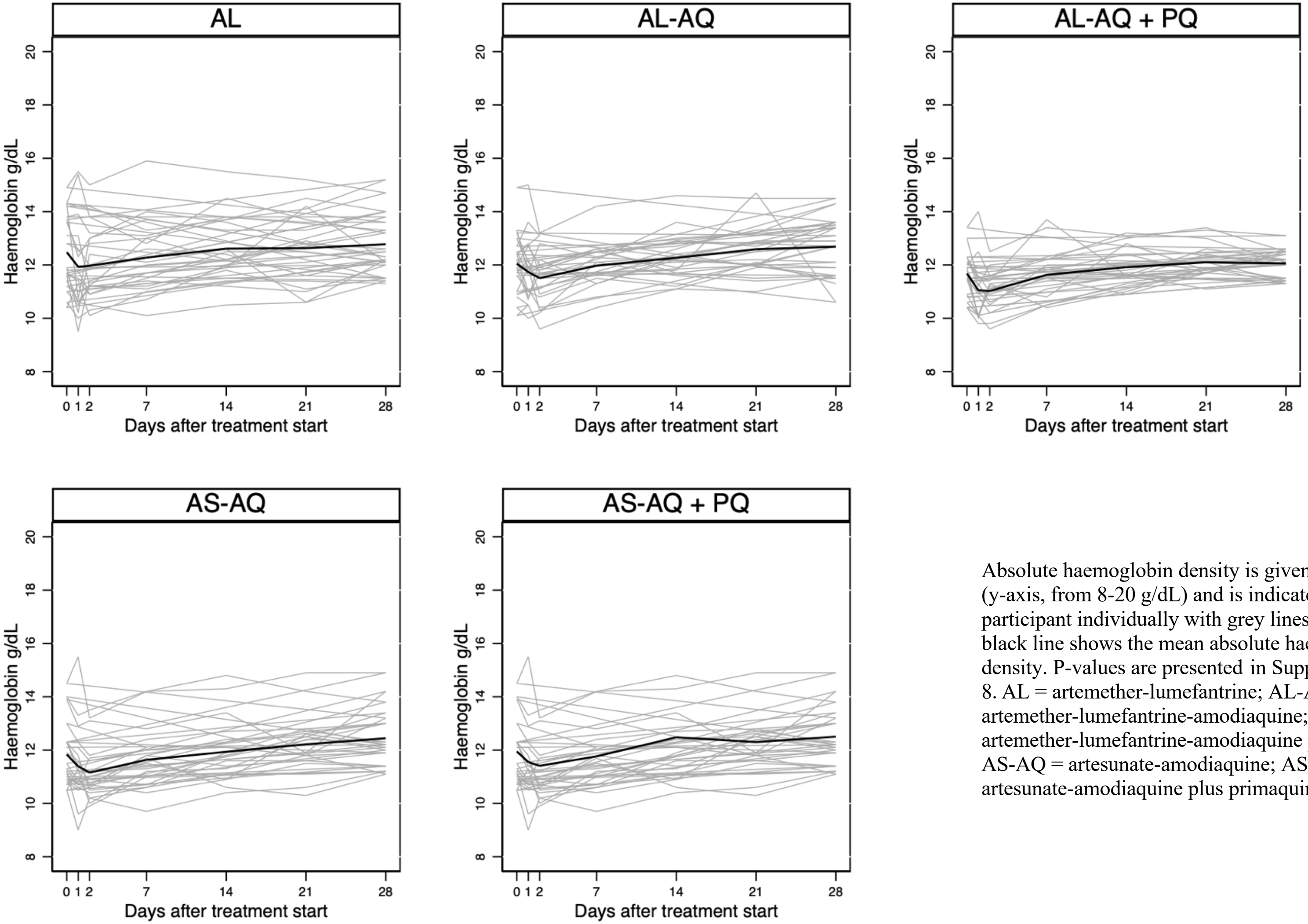

Absolute haemoglobin density is given in grams per dL (y-axis, from 8-20 g/dL) and is indicated for each participant individually with grey lines. The single black line shows the mean absolute haemoglobin density. P-values are presented in Supplementary table 8. AL = artemether-lumefantrine; AL-AQ = artemether-lumefantrine-amodiaquine; AL-AQ+PQ = artemether-lumefantrine-amodiaquine plus primaquine; AS-AQ = artesunate-amodiaquine; AS-AQ+PQ = artesunate-amodiaquine plus primaquine.

Supplementary Table 11. Biochemistry

Day of follow-up	Treatment arm	Mean ALT U/L (range)	p-value¥	p-value†	Mean AST U/L (range)	p-value¥	p-value†	Mean creatinine mg/dL (range)	p-value¥	p-value†
Day 0	**Overall**	23·45 (8-157)	·	·	29·24 (4-215)	·	·	0·64 (0·2-1·2)	·	·
	**AL**	20·90 (12-37)	*ref*	*ref*	23·95 (4-40)	*ref*	*ref*	0·63 (0·2-1·2)	*ref*	*ref*
	**AL-AQ**	19·15 (10-27)	*ref*	0·387	25·45 (13-35)	*ref*	0·562	0·77 (0·3-1·2)	*ref*	0·055
	**AL-AQ+PQ**	21·50 (8-67)	*ref*	0·881	30·30 (16-62)	*ref*	0·065	0·66 (0·2-1·2)	*ref*	0·650
	**AS-AQ**	25·50 (7-117)	*ref*	*ref*	27·25 (2-63)	*ref*	*ref*	0·58 (0·2-1·0)	*ref*	*ref*
	**AS-AQ+PQ**	24·75 (5-118)	*ref*	0·921	26·75 (6-42)	*ref*	0·881	0·66 (0·2-1·2)	*ref*	0·303
Day 2	**AL**	18·35 (9-40)	0·0694	*ref*	21·80 (4-40)	0·4851	*ref*	0·77 (0·1-1·4)	0·0280	*ref*
	**AL-AQ**	22·00 (8-57)	0·2813	0·125	28·37 (5-48)	0·1114	0·045	0·77 (0·2-1·3)	0·9404	0·279
	**AL-AQ+PQ**	29·16 (7-164)	0·2216	0·099	31·32 (11-94)	0·8206	0·226	0·61 (0·3-1·0)	0·3205	0·017
	**AS-AQ**	30·10 (12-126)	0·5847	*ref*	34·80 (8-163)	0·3167	*ref*	0·64 (0·3-1·0)	0·3306	*ref*
	**AS-AQ+PQ**	28·10 (7-105)	0·1422	0·821	28·90 (11-52)	0·5094	0·484	0·63 (0·2-1·2)	0·6319	0·573
Day 7	**AL**	20·40 (11-33)	0·7623	*ref*	24·55 (10-37)	0·8239	*ref*	0·61 (0·3-1·0)	0·6154	*ref*
	**AL-AQ**	22·00 (7-44)	0·1483	0·276	28·94 (8-44)	0·1364	0·143	0·74 (0·4-1·2)	0·6901	0·259
	**AL-AQ+PQ**	26·05 (8-129)	0·4681	0·380	29·84 (8-110)	0·8915	0·628	0·62 (0·4-0·8)	0·4581	0·915
	**AS-AQ**	26·45 (9-92)	0·8855	*ref*	27·30 (10-38)	0·9835	*ref*	0·65 (0·2-1·0)	0·2183	*ref*
	**AS-AQ+PQ**	25·20 (13-57)	0·9068	0·810	29·75 (12-49)	0·1975	0·293	0·72 (0·2-1·0)	0·1907	0·476
Day 14	**AL**	19·45 (10-38)	0·2991	*ref*	24·95 (9-39)	0·7192	*ref*	0·64 (0·2-1·0)	0·8152	*ref*
	**AL-AQ**	28·72 (10-157)	0·2054	0·137	36·17 (19-215)	0·2846	0·300	0·70 (0·2-1·0)	0·2615	0·861
	**AL-AQ+PQ**	25·11 (8-112)	0·6021	0·354	33·95 (15-86)	0·4069	0·140	0·60 (0·3-0·8)	0·3105	0·407
	**AS-AQ**	25·47 (15-65)	0·9046	*ref*	26·89 (4-42)	0·9360	*ref*	0·62 (0·2-1·2)	0·3620	*ref*
	**AS-AQ+PQ**	19·20 (9-30)	0·2683	0·043	25·05 (5-35)	0·3725	0·520	0·64 (0·4-1·2)	0·7263	0·742

Alanine aminotransferase (ALT), aspartate aminotransferase (AST) and creatinine were compared within treatment arms (p-value¥) using paired t-tests (with day 0 as reference) and between treatment arms (p-value†) using linear regression (adjusted for baseline levels). *Ref* = reference, · = not tested. AL = artemether-lumefantrine; AL-AQ = artemether-lumefantrine-amodiaquine; AL-AQ+PQ = artemether-lumefantrine-amodiaquine plus primaquine; AS-AQ = artesunate-amodiaquine; AS-AQ+PQ = artesunate-amodiaquine plus primaquine.

Supplementary Table 12. All adverse events

**Description**	**Total (n=100)**	**AL (n=20)**	**AL-AQ (n=20)**	**AL-AQ+PQ (n=20)**	**AS-AQ (n=20)**	**AS-AQ+PQ (n=20)**
Abdominal pain	23^{21}(5)	3^{3}	5^{5}(1)	4^{4}(1)	5^{5}(3)	6^{4}
Acute respiratory infection	26^{1}(17)	6(2)	2(2)	7(4)	6^{1}(4)	5(5)
Allergic contact dermatitis	1(1)	0	0	1(1)	0	0
Anemia	1^{1}	0	1^{1}	0	0	0
Asthenia	16^{16}(5)	2^{2}	4^{4}(2)	3^{3}	4^{4}(2)	3^{3}(1)
Chills	2^{2}	1^{1}	0	1^{1}	0	0
Conjunctivitis	3(3)	1(1)	1(1)	1(1)	0	0
Cough	9^{2}(4)	2^{1}(1)	1(1)	1	4^{1}(1)	1(1)
Diarrhea	4^{4}(1)	0	2^{2}(1)	0	1^{1}	1^{1}
Drowsiness	5^{5}(1)	1^{1}	1^{1}	2^{2}(1)	1^{1}	0
Dyspnea	1^{1}	0	0	1^{1}	0	0
Eczema	1(1)	0	1(1)	0	0	0
Elevation of ALT/ GPT	5^{2}(1)	0	1	2^{1}(1)	2^{1}	0
Elevation of ASAT/GOT	5^{1}	0	1	3^{1}	1	0
Elevation of creatinemia	3^{2}	2^{1}	0	0	0	1^{1}
Fatigue	4^{3}	2^{1}	0	2^{2}	0	0
Fever	2^{1}(1)	1^{1}(1)	0	0	0	1
Food indigestion	1(1)	0	0	0	1(1)	0
Headaches	51^{38}(23)	8^{7}(3)	10^{8}(5)	12^{10}(7)	8^{6}(4)	13^{7}(4)
Hyperleukocytosis	4^{1}	1	0	2^{1}	0	1
Leucopenia	3^{3}	0	0	0	2^{2}	1^{1}
Liquid diarrhea	1^{1}(1)	0	1^{1}(1)	0	0	0
Localized left arm pain	1	0	0	1	0	0
Loss of appetite	10^{10}	0	3^{3}	2^{2}	5^{5}	0
Low back pain	1	0	1	0	0	0
Muscular pain	10^{8}(1)	1(1)	1^{1}	3^{3}	3^{2}	2^{2}
Nausea	17^{17}(2)	3^{3}	3^{3}(1)	3^{3}	6^{6}(1)	2^{2}
Rhinitis	3(1)	1(1)	2	0	0	0
Rhinorrhea	13(1)	3	3	2	3	2(1)
Traumatic wound right food	1(1)	0	1(1)	0	0	0
Uncomplicated malaria	1(1)	1(1)	0	0	0	0
Vertigo	19^{18}(6)	2^{1}	3^{3}(1)	6^{6}(4)	4^{4}(1)	4^{4}
Vomiting	15^{14}(3)	4^{4}(1)	4^{3}(1)	3^{3}	3^{3}(1)	1^{1}
**ALL**	*262^{172}*	*45^{26}*	*52^{35}*	*62^{43}*	*59^{42}*	*44^{26}*
MILD	*181^{129}*	*33^{21}*	*33^{22}*	*42^{32}*	*41^{30}*	*32^{24}*
MODERATE	*81^{43}*	*12^{5}*	*19^{13}*	*20^{11}*	*18^{12}*	*12^{2}*

85/100 participants experienced a total of 262 adverse events over the course of the trial; 181 categorised for severity by the study clinician (in accordance with the study protocol and data safety and monitoring charter) as 'mild' and 81 as 'moderate'. No severe adverse events or serious adverse events (SAE) occurred during the trial. The frequency of all AEs is given outside parentheses, with the frequency of moderate AEs in parentheses. The frequency of AEs that were related to drug treatment (defined as probably, possibly or definitely related to treatment) is given in superscript. 172 of the 262 AEs were classified as possibly, probably or definitely related to the study drug; of these, 129/169 were mild and 43/169 were moderate.

References

1. Dicko A, Roh ME, Diawara H, et al. Efficacy and safety of primaquine and methylene blue for prevention of Plasmodium falciparum transmission in Mali: a phase 2, single-blind, randomised controlled trial. Lancet Infect Dis. 2018 Jun;18(6):627–39.

2. Dicko A, Brown JM, Diawara H, et al. Primaquine to reduce transmission of Plasmodium falciparum malaria in Mali: a single-blind, dose-ranging, adaptive randomised phase 2 trial. Lancet Infect Dis. 2016 Jun;16(6):674–84.

3. Stone W, Mahamar A, Sanogo K, et al. Pyronaridine–artesunate or dihydroartemisinin–piperaquine combined with single low-dose primaquine to prevent Plasmodium falciparum malaria transmission in Ouélessébougou, Mali: a four-arm, single-blind, phase 2/3, randomised trial. Lancet Microbe. 2022 Jan;3(1):e41–51.

4. Meerstein-Kessel L, Andolina C, Carrio E, et al. A multiplex assay for the sensitive detection and quantification of male and female Plasmodium falciparum gametocytes. Malar J. 2018 Dec;17(1):441.

5. Bousema T, Okell L, Shekalaghe S, et al. Revisiting the circulation time of Plasmodium falciparum gametocytes: molecular detection methods to estimate the duration of gametocyte carriage and the effect of gametocytocidal drugs. 2010;11.

6. Méndez F, Muñoz Á, Plowe CV. Use of area under the curve to characterize transmission potential after antimalarial treatment. Am J Trop Med Hyg. 2006 Oct 1;75(4):640–4.
